# Molecular Docking Identifies 1,8-Cineole (Eucalyptol) as A Novel PPARγ Agonist That Alleviates Colon Inflammation

**DOI:** 10.3390/ijms24076160

**Published:** 2023-03-24

**Authors:** Balaji Venkataraman, Saeeda Almarzooqi, Vishnu Raj, Bhoomendra A. Bhongade, Rajesh B. Patil, Veedamali S. Subramanian, Samir Attoub, Tahir A. Rizvi, Thomas E. Adrian, Sandeep B. Subramanya

**Affiliations:** 1 Department of Physiology, College of Medicine and Health Sciences, United Arab Emirates University, Al Ain P.O. Box 15551, United Arab Emirates; 2 Zayed Bin Sultan Center for Health Sciences, College of Medicine and Health Sciences, United Arab Emirates University, Al Ain P.O. Box 15551, United Arab Emirates; 3 Department of Pathology, College of Medicine and Health Sciences, United Arab Emirates University, Al Ain P.O. Box 15551, United Arab Emirates; 4 Department of Pharmaceutical Chemistry, RAK College of Pharmacy, RAK Medical & Health Sciences University, Ras Al Khaimah P.O. Box 11172, United Arab Emirates; 5 Department of Pharmaceutical Chemistry, Sinhgad College of Pharmacy, Vadgaon (BK), Pune 411 041, India; 6 Department of Medicine, University of California, Irvine, CA 92697, USA; 7 Department of Pharmacology and Therapeutics, College of Medicine and Health Sciences, United Arab Emirates University, Al Ain P.O. Box 15551, United Arab Emirates; 8 Department of Medical Microbiology and Immunology, College of Medicine and Health Sciences, United Arab Emirates University, Al Ain P.O. Box 15551, United Arab Emirates; 9 Department of Basic Medical Sciences, College of Medicine, Mohammed Bin Rashid University of Medicine and Health Sciences, Dubai P.O. Box 505055, United Arab Emirates

**Keywords:** 1,8-cineole (eucalyptol), DSS-colitis, PPARγ, IBD

## Abstract

Inflammatory bowel disease, comprising Crohn’s disease (CD) and ulcerative colitis (UC), is often debilitating. The disease etiology is multifactorial, involving genetic susceptibility, microbial dysregulation, abnormal immune activation, and environmental factors. Currently, available drug therapies are associated with adverse effects when used long-term. Therefore, the search for new drug candidates to treat IBD is imperative. The peroxisome proliferator-activated receptor-γ (PPARγ) is highly expressed in the colon. PPARγ plays a vital role in regulating colonic inflammation. 1,8-cineole, also known as eucalyptol, is a monoterpene oxide present in various aromatic plants which possess potent anti-inflammatory activity. Molecular docking and dynamics studies revealed that 1,8-cineole binds to PPARγ and if it were an agonist, that would explain the anti-inflammatory effects of 1,8-cineole. Therefore, we investigated the role of 1,8-cineole in colonic inflammation, using both in vivo and in vitro experimental approaches. Dextran sodium sulfate (DSS)-induced colitis was used as the in vivo model, and tumor necrosis factor-α (TNFα)-stimulated HT-29 cells as the in vitro model. 1,8-cineole treatment significantly decreased the inflammatory response in DSS-induced colitis mice. 1,8-cineole treatment also increased nuclear factor erythroid 2-related factor 2 (Nrf2) translocation into the nucleus to induce potent antioxidant effects. 1,8-cineole also increased colonic PPARγ protein expression. Similarly, 1,8-cineole decreased proinflammatory chemokine production and increased PPARγ protein expression in TNFα-stimulated HT-29 cells. 1,8-cineole also increased PPARγ promoter activity time-dependently. Because of its potent anti-inflammatory effects, 1,8-cineole may be valuable in treating IBD.

## 1. Introduction

Inflammatory bowel disease (IBD) encompasses Crohn’s disease and ulcerative colitis. Crohn’s causes transmural ulcers of any section of the gastrointestinal tract (GI), most commonly in the terminal ileum and colon, while ulcerative colitis (UC) is characterized by inflammation localized to the colon [1]. The etiology of IBD is multifactorial, including genetic susceptibility, aberrant stimulation of the immune system, gut microbiota dysbiosis, and environmental factors [1]. The prevalence of IBD is increasing worldwide, including in the Middle Eastern region [2]. The currently available drug therapy is often associated with marked side effects. Therefore, the search for new drug candidates is an active area of research.

Peroxisome proliferator-activated receptors (PPARs), a group of nuclear receptor proteins involved in lipid metabolism, cell proliferation, and insulin sensitivity, also play a critical role in regulating inflammation [2]. PPARs exist in three isoforms: PPARα, PPARβ/δ, and PPARγ. PPARγ is present in three different variants, PPARγ1, 2, and 3, which are created by alternative splicing from the same gene. PPARγ1 and PPARγ3 encode for identical proteins. PPARγ1 is expressed ubiquitously, whereas PPARγ3 is substantially expressed exclusively in adipose tissue, colonic mucosa, and macrophages [3].

PPARs heterodimerize with retinoid X receptors (RXR), and this complex then binds to specific regions of DNA called the PPAR response elements (PPRE), to activate the transcription of target genes. PPARγ regulates cell proliferation in various other tissues and organs, including the colon, breast, prostate, and bladder. PPARγ signaling dysregulation is associated with tumor growth in these organs [4]. PPARγ promotes the inactivation of NF-κB during the inflammatory response. NF-κB is a key regulator that fuels inflammation. The inactivation step may include direct binding to NF-κB or ubiquitination leading to the proteolytic destruction of NF-κB^p65^. PPARγ also has an indirect influence on NF-κB expression. PPARγ also increases the expression of antioxidant enzymes such as catalase, superoxide dismutase, and heme oxygenase-1, leading to a decrease in reactive oxygen species (ROS), which are secondary mediators of the inflammatory response [5]. Therefore, PPARγ plays a critical role in regulating inflammatory and antioxidant responses.

1,8-cineole (also known as eucalyptol) is a monoterpenoid oxide present in the essential oil form of the plant families of *Myrtaceae*, *Lamiaceae*, and *Zingiberaceae*. 1,8-cineole administration has been shown to have clinical benefits in patients with asthma and COPD due to its potent anti-inflammatory and antioxidant effects [6,7]. In a recent study, 1,8-cineole significantly reduced NF-κB^p65^ phosphorylation in a PPARγ-dependent manner in LPS-induced vascular endothelium dysfunction [8]. 1,8-cineole also protected colonic damage in TNBS-induced colitis in rats [9,10]. However, the molecular mechanisms responsible for the protective effects of 1,8-cineole on colonic damage have not yet been characterized. On the basis of the literature, as mentioned above, we docked 1,8-cineole to the PPARγ protein and conducted molecular dynamics investigations to shed light on this interaction. Subsequently, we evaluated the anti-inflammatory effects of 1,8-cineole as a PPARγ agonist, using in vivo and in vitro models of colonic inflammation.

## 2. Results

### 2.1. Molecular Docking Studies

**Figure 1:**
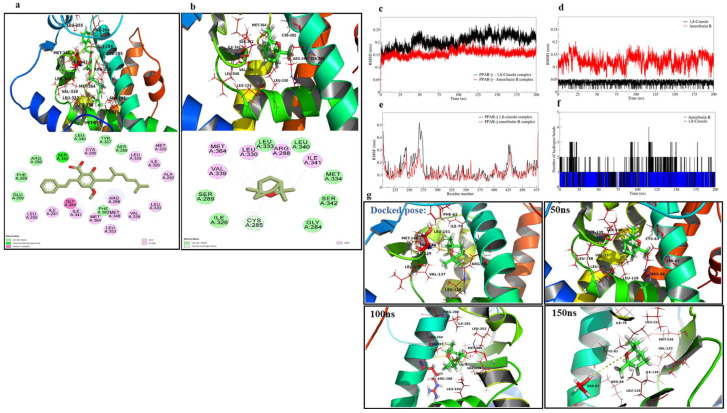
Molecular docking and dynamics of 1,8-cineole with PPARγ. The docked pose and the interactions at the binding site of PPARγ. (**a**) Interactions of amorfrutin B and (**b**) interactions of 1,8-cineole. The analysis of the MD simulation (**c**) RMSD in PPARγ backbone atoms, (**d**) RMSD in 1,8-cineole atoms and amorfrutin B atoms, (**e**) RMSF in PPARγ residues, (**f**) hydrogen bond analysis for 1,8-cineole and amorfrutin B, (**g**) The analysis of trajectories extracted at different time intervals (50–150 ns) of the MD simulations.

The ligand amorfrutin B, when docked to the binding site of PPARγ, was found to have interactions with Ser342, Ile341, Gly284, Leu255, Ile281, Arg288, Met329, Leu330, Ile326, Ala292, Leu333, Met348, Leu353, Val339, Met364, and Cys285 of PPARγ (Figure 1a). The docked pose of amorfrutin B almost matched the co-crystallized pose, and the root-mean-square deviation (RMSD) of the atoms of both poses was found to be below 2 Å. The optimized structure of 1,8-cineole was also docked to the binding site of PPARγ. The best-docked pose showed that the lipophilic part of 1,8-cineole occupying the hydrophobic binding cavity of PPARγ was surrounded by Val339, Cys285, Leu330, and Arg288 (Figure 1b). The docking results are given in Table 1.

The stability of the resultant systems of 1,8-cineole and amorfrutin B bound to PPARγ was investigated with 200 ns extended MD simulations. The analysis of the trajectories of each system for RMSD in PPAR-γ backbone atoms showed deviations until the 100 ns simulation period with 1,8-cineole, which was reasonably stabilized thereafter until the end of the simulation, with an average RMSD of 0.2034 ± 0.0205 nm (Figure 1c). Meanwhile, the RMSD in PPARγ backbone atoms in the apoenzyme was 0.2184 ± 0.03296 nm (Figure S1), similar to PPARγ in complex to 1,8-cineole. Meanwhile, the RMSD in PPAR- γ backbone atoms with amorfrutin B was reasonably stabilized after around 25 ns of simulation with an average RMSD of 0.1547 ± 0.110 nm. The RMSD analysis of the ligand atom (Figure 1d), for which the average RMSD for 1,8-cineole is 0.03732 ± 0.0077 nm, is far lower than for amorfrutin B with an average of 0.12306 ± 0.020 nm. The RMSF analysis showed that the binding site residues ranging from 240 to 275 underwent major fluctuations in the case of 1,8-cineole bound to PPARγ, while the other system with amorfrutin B bound had comparably fewer fluctuations (Figure 1e). The RMSF in apo-PPARγ showed similar fluctuations but lesser magnitude (Figure S2) compared to 1,8-cineole-bound PPARγ. Hydrogen bond analysis showed a maximum of three hydrogen bonds (except in one instance of four hydrogen bonds) being formed with amorfrutin B, out of which one hydrogen bond was consistently formed throughout the MD simulation Figure 1f). The hydrogen bond between the 1,8-cineole oxygen atom and Ser140 residue was observed until 50 ns. At around 100 ns, a new hydrogen bond with Arg86 and thereafter, until the end of the simulation period, a hydrogen bond with Ser87 was observed (Figure 1g).

### 2.2. Effect of 1,8-Cineole on the Disease Activity Index (DAI), Colonic Length, and Myeloperoxidase (MPO) Activity

**Figure 2:**
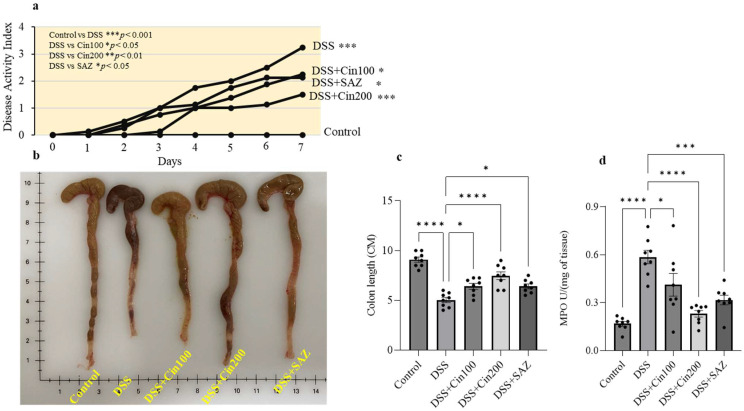
Effect of 1,8-cineole on the disease activity index (DAI), colonic length, and myeloperoxidase (MPO) activity. DSS administration significantly (**a**) increased the DAI, (**b**,**c**) decreased colonic length, and (**d**) increased MPO activity. 1,8-cineole treatment significantly prevented (**a**) the increase in DAI, (**b**,**c**) the decrease in colonic length, and (**d**) the increase in MPO enzyme activity. Data were obtained from n = 8 animals in each group. The results are expressed as the means ± SEM. * *p* ≤ 0.05, ** *p* ≤ 0.01, *** *p* ≤ 0.001, and **** *p* ≤ 0.0001.

DSS administration significantly increased the DAI and MPO enzyme activity and shortened the colonic length. 1,8-cineole treatment markedly reduced this rise in DAI and MPO activity, indicating its protective role (Figure 2a,d). It is well-known that DSS administration shortens colonic length due to fibrosis. 1,8-cineole treatment also significantly prevented the shortening of the colonic length (Figure 2b,c). Sulfasalazine, a clinically used drug, was also evaluated along with 1,8-cineole as a positive control. The higher dose (200 mg/kg body weight) of 1,8-cineole conferred better protection in terms of DAI, MPO activity, and colonic length than sulfasalazine.

### 2.3. Effect of 1,8-Cineole on Colonic Histology

**Figure 3:**
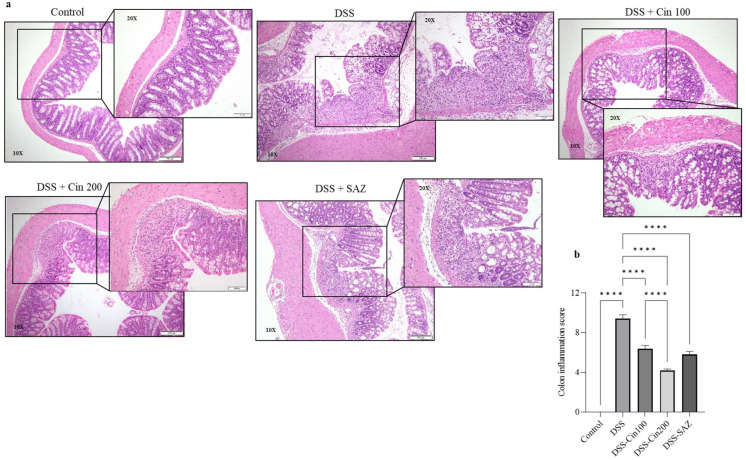
Effect of 1,8-cineole on colon histology. (**a**,**b**) DSS administration significantly increased the colonic inflammation score and crypt aberration. 1,8-cineole treatment significantly prevented the increase in the colonic inflammation score and protected against crypt aberration. Data were obtained from n = 6 animals per group. The results are expressed as the means ± SEM. **** *p* ≤ 0.0001.

DSS administration dramatically altered the colonic microarchitecture. The colonic epithelium was depleted by DSS administration with focal necrosis. Enhanced neutrophil infiltration was observed in the lamina propria and submucosal compartment, along with crypt aberration. The colonic inflammation score was significantly increased in the DSS-administered group compared to the controls. The 1,8-cineole treatment in the DSS-administered group significantly protected the epithelial architecture, and decreased focal necrosis was observed (Figure 3a). 1,8-cineole treatment also decreased the neutrophil infiltration, contributing to a significantly decreased colonic inflammation score (Figure 3b) and decreased crypt aberration. These results indicate that 1,8-cineole treatment significantly protected the colonic microarchitecture.

### 2.4. Effect of 1,8-Cineole on Proinflammatory Cytokine Protein and mRNA

**Figure 4:**
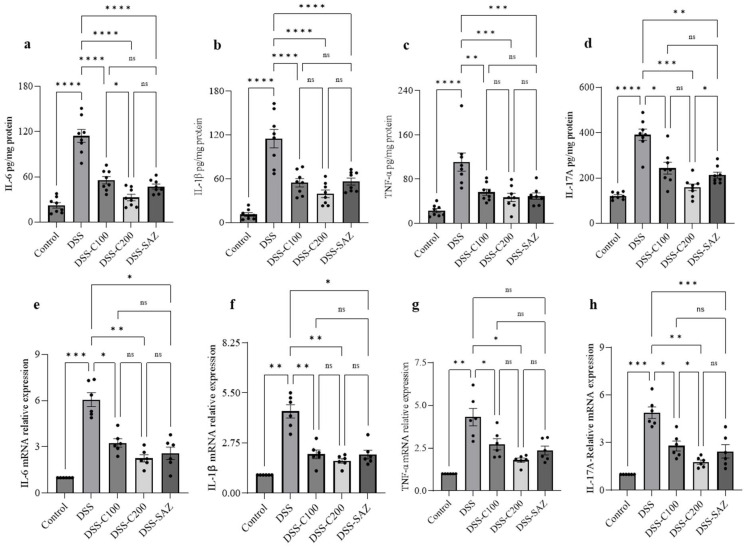
Effect of 1,8-cineole on proinflammatory cytokine protein and mRNA expression. (**a**–**d**) DSS administration significantly increased colonic proinflammatory cytokine levels. 1,8-cineole treatment significantly prevented the increase in proinflammatory cytokines. (**e–h**) Similarly, DSS administration significantly increased the expression of proinflammatory cytokine mRNA levels. 1,8-cineole treatment significantly prevented this increase in expression of proinflammatory cytokine mRNA. Data were obtained from n = 8 animals per group. The results are expressed as the means ± SEM. * *p* ≤ 0.05, ** *p* ≤ 0.01, *** *p* ≤ 0.001, **** *p* ≤ 0.0001, and ns indicates results that were not significant.

To investigate the proinflammatory cytokines involved in colitis, the DSS-administered colon and 1,8-cineole-treated colon tissue were homogenized and ELISA was performed to estimate tissue proinflammatory cytokine levels. Similarly, a piece of colon tissue from the different experimental groups was subjected to mRNA analysis using real-time PCR to measure the change in the mRNA expression levels. DSS administration significantly increased levels of the proinflammatory cytokines, IL-6, IL-1β, TNF-α, and IL17A at the protein level. 1,8-cineole treatment significantly prevented the increases in these proinflammatory cytokines (Figure 4a–d). Similarly, DSS administration increased proinflammatory cytokine mRNA expression, which was prevented by 1,8-cineole treatment (Figure 4e–h). These results indicate that 1,8-cineole treatment inhibits proinflammatory cytokine levels in both protein and mRNA expression, confirming its potent anti-inflammatory action.

### 2.5. Effect of 1,8-Cineole on COX2 and iNOS Protein, mRNA, and Tissue Nitrite Levels

**Figure 5:**
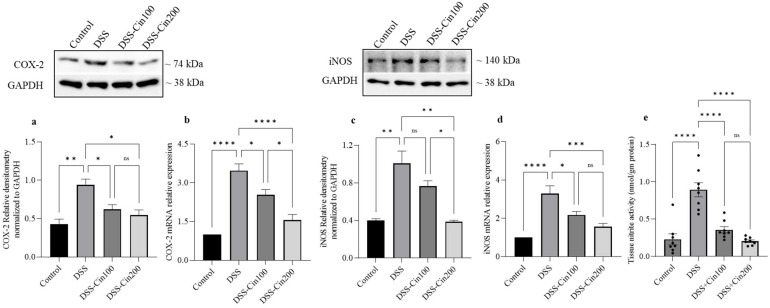
Effect of 1,8-cineole on COX2 and iNOS protein and mRNA expression as well as tissue nitrite levels. (**a**,**c**) The upper panel shows COX2 and iNOS protein expression by Western blot. A representative of protein bands that were normalized to GAPDH protein as an internal control is shown. Densitometry analysis shows that DSS administration significantly increased (**a**) COX2, (**c**) iNOS protein, and (**b**,**d**) mRNA expression. In contrast, 1,8-cineole treatment significantly prevented the increases in protein and mRNA expression. (**a**–**d**) DSS treatment increased tissue nitrite levels, while (**e**) 1,8-cineole treatment prevented this. Data were obtained from n = 4 animals/group. The results are expressed as the means ± SEM. * *p* ≤ 0.05, ** *p* ≤ 0.01, *** *p* ≤ 0.001, **** *p* ≤ 0.0001, and ns indicates results that were not significant.

When the development of colitis increases under the influence of proinflammatory cytokines, increases in the expression of COX2 and iNOS, two powerful proinflammatory mediators, are also seen. Therefore, we investigated the effect of 1,8-cineole on the expression of COX2, iNOS protein, and mRNA, as well as tissue nitrite levels. DSS administration significantly increased COX2 and iNOS expression at both the protein and mRNA levels. 1,8-cineole treatment prevented these increases in COX2 and iNOS protein and mRNA (Figure 5a–d). The induction of iNOS due to inflammation increased tissue nitric oxide levels. Increased nitric oxide interacts with superoxide radicals to generate cytotoxic peroxynitrite. DSS administration significantly increased tissue nitrite levels and 1,8-cineole treatment significantly prevented its formation, indicating its ability to inhibit iNOS (Figure 5e).

### 2.6. Effect of 1,8-Cineole on Keap1 and Nrf2 System and Antioxidant Enzymes

**Figure 6:**
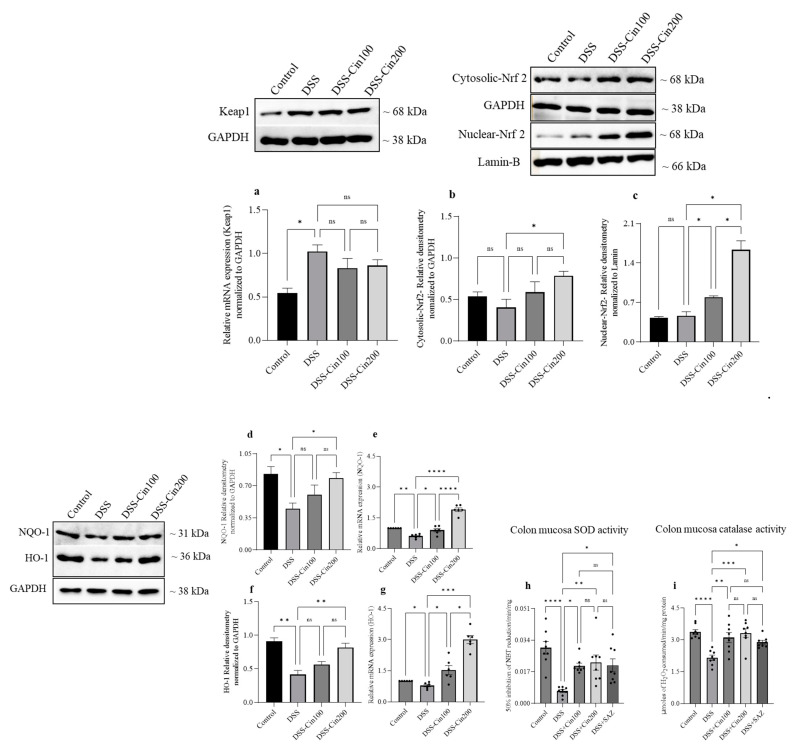
Effect of 1,8-cineole on Keap1, Nrf2, HO1, NQO1, superoxide dismutase (SOD), and catalase (CAT) activity. The upper panel shows (**a**) Keap1 and (**b**,**c**) cytosolic Nrf2 and nuclear Nrf2 protein expression. Representative protein bands were normalized to GAPDH and lamin proteins as the internal controls as shown. Densitometric analysis showed that DSS administration increased Keap1 protein expression while 1,8-cineole did not further increase Keap1 protein expression. Nuclear Nrf2 protein expression was markedly increased by 1,8-cineole treatment compared to the DSS-administered group. (**c**) DSS administration significantly decreased (**d**) NQO1, (**f**) HO1 protein, and (**e**,**g**) mRNA expression, while 1,8-cineole treatment prevented these decreases. DSS administration significantly decreased (**h**) colon tissue SOD and (**i**) CAT enzyme activity, while 1,8-cineole treatment restored their activities toward the control levels. Data were obtained from n = 4 for protein, n = 6 for mRNA, and n = 8 animals for SOD and CAT enzyme activity; the results are expressed as the means ± SEM. * *p* ≤ 0.05, ** *p* ≤ 0.01, *** *p* ≤ 0.001, **** *p* ≤ 0.0001, and ns indicates results that were not significant.

An increase in oxidative stress is harmful to tissues. The nuclear factor erythroid 2-related factor 2 (Nrf2) and its inhibitor Kelch-like ECH-associated protein-1 (Keap1) axis plays an important role in this. Under normal conditions, Nrf2 and Keap1 are located in the cytoplasm, while under stressful conditions, Nrf2 separates from the Keap1 regulator protein and translocates to the nucleus. In the nucleus, Nrf2 activates the expression of various detoxifying systems to mitigate oxidative stress. DSS administration did not affect the cytoplasmic retention of Nrf2 but increased Keap1 protein expression. 1,8-cineole treatment increased Nrf2 nuclear translocation but did not affect Keap1 expression. (Figure 6a–c). The nuclear fraction of Nrf2 activates an antioxidant responsive element to increase the transcription of key detoxifying enzymes such as NAD(P)H:quinone oxidoreductase (NQO1) and stress-responsive proteins such as heme oxygenase-1 (HO-1). 1,8-cineole increased both NQO1 and HO1 protein and mRNA expression along with an increase in superoxide dismutase-1 (SOD-1) and catalase enzyme activity (Figure 6d–i).

### 2.7. Effect of 1,8-Cineole on PPARγ, PPARα, PPARβ/δ, and Phospho-Nf-κB (p65) Protein Expression

**Figure 7:**
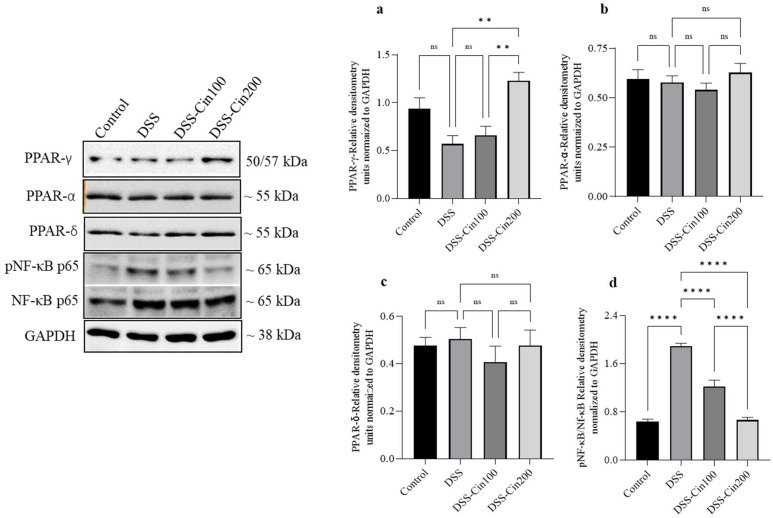
Effect of 1,8-cineole on PPARγ, PPARα, PPARδ, and pNFκB/NFκB protein expression. (**a**) The side panel shows PPARγ, PPARα, PPARδ, and pNFκB/NFκB protein expression. Protein bands were normalized to the GAPDH protein internal control as shown. DSS administration significantly (**b**) decreased PPARγ expression and 1,8 cineole at the higher concentration (**b**) increased it above the control. DSS administration and 1,8-cineole treatment (**c**,**d**) did not alter PPARα and PPARδ expression. DSS administration markedly increased the phosphorylation of NFκB, while 1,8-cineole treatment at the higher concentration prevented this. Data were obtained from n = 4 animals. The results are expressed as the means ± SEM. ** *p* ≤ 0.01, and ns indicates results that were not significant.

We subsequently investigated the effect of 1,8-cineole on the PPARs protein and phosphorylation of Nf-κB protein expression. DSS administration decreased PPARγ protein expression. 1,8-cineole treatment increased PPARγ expression (Figure 7a). Neither DSS administration nor 1,8-cineole treatment affected PPARα or PPARδ protein expression (Figure 7b,c). DSS administration markedly increased the phosphorylation of Nf-κB (p65) protein expression and 1,8-cineole administration at the higher concentration prevented this (Figure 7d).

### 2.8. Effect of 1,8-Ccineole on HT-29 Cell Viability, IL-8, CXCL-1 Chemokine mRNA Expression, and PPARγ Promoter Activation

**Figure 8:**
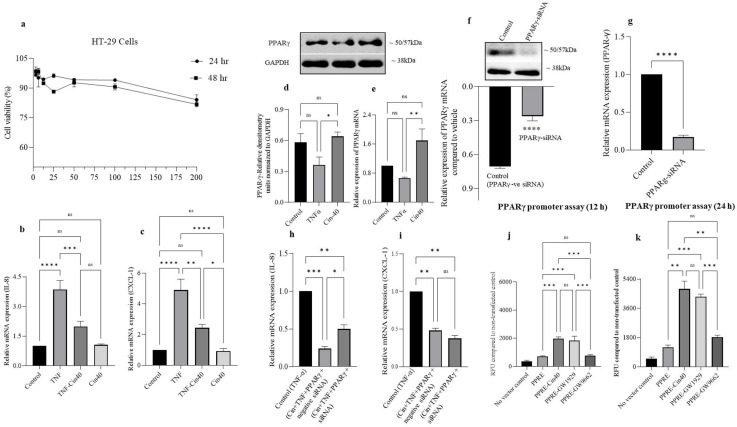
Effect of 1,8-cineole on HT-29 cell viability, proinflammatory chemokine mRNA expression, PPARγ expression, and PPARγ promoter assay. (**a**) 1,8-cineole treatment did not affect HT-29 cell viability up to a 200 μm concentration. (**b**,**c**) 1,8-cineole significantly downregulated IL8 and CXCL1 mRNA expression in TNF-α-challenged HT-29 cells. (**d**,**e**) 1,8-cineole significantly increased PPARγ protein and mRNA expression. (**f**,**g**) PPARγ siRNA significantly downregulated PPARγ protein and mRNA expression. (**h**) PPARγ siRNA transfection downregulated IL8 mRNA expression, and 1,8-cineole treatment partially prevented this. (**i**) siRNA transfection did not affect CXCL1 mRNA expression in the presence of 1,8-cineole. (**j**,**k**) 1,8-cineole treatment significantly enhanced PPARγ promoter activation time-dependently (12 and 24 h). The PPARγ activator (GW1929) and PPARγ inhibitor (GW9662) worked as expected. * *p* ≤ 0.05, ** *p* ≤ 0.01, *** *p* ≤ 0.001, ****p ≤ 0.0001, and ns indicate results that were not significant.

HT-29 cells are colon adenocarcinoma cells that robustly express PPARγ proteins. Therefore, we used TNF-α (1 ng/mL)-challenged HT-29 cell as an in vitro model of colonic inflammation to investigate the role of 1,8-cineole on PPARγ and the inflammatory response. In our initial experiments, we determined the non-cytotoxic dose of 1,8-cineole by exposing different concentrations to HT-29 cells for 24 and 48 h. 1,8-cineole did not affect HT-29 cell viability at the highest concentration of 200 μM at 24 and 48 h (Figure 8a). Based on these results, we selected a dose of 40 μM to test the effect of 1,8-cineole on proinflammatory chemokines such as IL-8 and CXCL-1 mRNA expression in TNF-α stimulated HT-29 cells. TNF-α stimulation significantly increased IL-8 and CXCL-1 mRNA expression and 1,8-cineole treatment partially prevented this increase. However, 1,8-cineole alone (40 μM) did not affect either IL-8 or CXCL-1 mRNA expression (Figure 8b,c). Next, we investigated the effect of 1,8-cineole treatment on PPARγ protein and mRNA expression. 1,8-cineole significantly increased PPARγ protein and mRNA expression for the TNF-α treated condition (Figure 8d,e). Subsequently, we investigated the effect of PPARγ knockdown on chemokine expression using siRNA. The PPARγ siRNA transfection resulted in a significant decrease in PPARγ protein and mRNA expression (Figure 8f,g). Next, we investigated whether inhibiting PPARγ would affect IL-8 and CXCL-1 chemokine mRNA expression in TNF-α stimulated HT-29 cells in the presence of 1,8-cineole. 1,8-cineole treatment in the absence of PPARγ protein inhibition (Cin40+TNF-α+PPARγ negative siRNA) further decreased IL-8 mRNA expression compared to uninhibited PPARγ (Figure 8h (Cin40+TNF-α+PPARγ siRNA)), suggesting that the anti-inflammatory action of 1,8-cineole’s action is probably mediated via PPARγ proteins. However, the knockdown of PPARγ proteins did not affect CXCL-1 mRNA expression in the presence of 1,8-cineole (Figure 8i). Furthermore, we also investigated the role of 1,8-cineole on PPARγ promoter expression. A full-length promoter was transfected into HT-29 cells, followed by 1,8-cineole treatment. 1,8-cineole significantly increased PPARγ promoter time-dependently (12 and 24 h) (Figure 8j,k). These results indicate that 1,8-cineole mediates its anti-inflammatory action through the PPARγ protein, which is transcriptionally regulated.

## 3. Discussion

In the current study, we investigated the effect of 1,8-cineole on colonic inflammation using both in vivo and in vitro models. Based on previous reports, 1,8-cineole exerts anti-inflammatory effects on the vascular endothelium via PPARγ activation. Therefore, our initial approach was to understand the interaction of 1,8-cineole with PPARγ proteins, by simulating the docking and dynamics studies and comparing these interactions with recently characterized PPARγ agonist amorfrutin B. The optimized structure of amorfrutin B was docked to the binding site to validate the flexible docking protocol. The interactions produced by the docked pose of amorfrutin B are similar to those produced by the co-crystallized pose of 1,8-cineole, a monoterpene bicyclic ether. Though the binding energy estimate was slightly higher in the case of 1,8-cineole (−5.7 kcal/mol) than the amorfrutin B (−6.9 kcal/mol), the interaction pattern suggests that 1,8-cineole also fit well in the binding site and produced the critical hydrophobic interactions. No hydrogen bonds were formed with the docked pose of 1,8-cineole.

The analysis of RMSD in backbone atoms in 1,8-cineole- and amorfrutin B-bound PPARγ complexes suggested both ligands’ stability and good binding affinity as the average RMSD is below 0.2 nm. In the case of RMSD in ligand atoms, it was found that the 1,8-cineole molecule, having a rigid structure, no rotatable bonds, and a possibly limited number of conformational flexibilities, had a lower RMSD compared to amorfrutin B. Amorfrutin B could adopt different conformations, which is evident from the deviations in the RMSD in ligand atoms. The RMSF analysis suggested that the major fluctuations in the binding site residues ranging from 240 to 275 in the case of 1,8-cineole bound to PPARγ may be attributed to the small and rigid molecular size of 1,8-cineole. The side chains of the residues in the unoccupied space in the binding cavity when 1,8-cineole is bound may undergo such fluctuations. On the other hand, the amorfrutin B-bound system had comparably fewer fluctuations. Contrary to the docking results, 1,8-cineole made a stable hydrogen bond with residues Ser140 or Arg86, or Ser87. Overall, the MD simulation results of 1,8-cineole suggest that 1,8-cineole remains bound stably at the binding cavity with good binding affinity.

We employed a DSS-induced mouse model of colitis as an in vivo model to evaluate the anti-inflammatory effects of 1,8-cineole on colonic inflammation. 1,8-cineole decreased the disease activity index (DAI) and significantly prevented colonic shortening along with decreases in MPO enzyme activity. Histological changes were also prevented by 1,8-cineole, with less inflammation, protected surface epithelial structure integrity, and less crypt aberration mediated by DSS. In a previous study, it was shown that 1,8-cinole decreased MPO activity in trinitrobenzene sulfonic acid (TNBS)-induced colitis in rats. However, no other inflammatory parameters were evaluated in that study.

1,8-cineole significantly decreased proinflammatory cytokine levels, including IL-6 IL-1β, TNF-α, and IL17A. The ability of 1,8-cineole to suppress proinflammatory cytokines has also been demonstrated in previous studies where 1,8-cineole treatment was effective in reducing proinflammatory cytokines in LPS-induced lung inflammation, human mononuclear cells, and lung macrophages [11,12,13]. 1,8-cineole treatment also reduced the severity of vascular endothelial inflammation, mediated by proinflammatory cytokines in LPS-induced systemic inflammation in mice [14]. Additionally, 1,8-cineole reduced the expression of TNF-α and IL-1β mRNA in human umbilical vein endothelial cells stimulated by LPS [15]. A double-blind, placebo-controlled trial on patients with bronchial asthma also reported that eucalyptol (1,8-cineole) inhibited proinflammatory cytokines [16]. The IL-17A inflammatory cytokine is elevated in the colonic mucosa of patients with ulcerative colitis [17]. In DSS-induced colitis in experimental mice, IL-17A is critical in regulating inflammation [18]. 1,8-cineole treatment also reduced IL-17A protein and mRNA expression in our study. The ability of 1,8-cineole to reduce IL-17A expression was previously demonstrated in a mouse model of asthma [19]. 1,8-cineole also inhibited the expression of the proinflammatory mediators COX2 and iNOS at the protein and mRNA levels. In a recent study, treatment with the essential oil of *Cinnamomum insularimontanum* and *Lavandula viridis* L’Hér, which contain approximately 36% 1,8-cineole, reduced macrophage COX2 and iNOS levels [20,21]. These reports indicate that 1,8-cineole possesses potent anti-inflammatory properties that may be beneficial in treating either acute or chronic inflammatory conditions.

The nuclear factor erythroid 2-related factor 2 (Nrf2)/Kelch-like ECH-associated protein 1 (Keap1) system protects cells from oxidative stress and inflammation. It is a key regulator of cellular protective responses to endogenous and exogenous stressors brought on by reactive oxygen species [22]. Nrf2 controls inflammation and tissue damage in IBD [23]. The expression of the HMOX1 gene, which encodes heme oxygenase 1 (HO-1) and the NQO1 genes, is critical for protection against oxidative stress and chronic inflammation [24,25]. Keap1 is inactivated under oxidative stress by the modification of its cysteine residues. This results in the accumulation and nuclear translocation of Nrf2 proteins. Once Nrf2 is translocated into the nucleus, it binds to the antioxidant responsive element (ARE) to transcribe its target genes (HO-1, NQO1, and SOD) to provide protection against oxidative insults [26]. We observed that 1,8-cineole treatment resulted in the nuclear translocation of Nrf2 and increased HO1, NQO1 protein, and mRNA expression, along with concomitant decreases in SOD and CAT activity. These results indicate that the antioxidant activity of 1,8-cineole is mediated by activating the Nrf2/Keap1 system. Previous studies have reported that 1,8-cineole protected liver cells from bisphenol A-induced and acetaminophen-induced liver injury by activating the Nrf2/Keap1 system [27,28]. 1,8-cineole also increased SOD expression through the nuclear translocation of Nrf2, which protected pheochromocytoma cells from hydrogen peroxide-mediated injury [29]. Thus, mounting evidence indicates that 1,8-cineole possesses potent antioxidant properties, mediated by the activation of the Nrf2/Keap1 pathway.

PPARγ is a nuclear receptor superfamily member and one of the most studied ligand-inducible transcription factors. PPARγ involvement is best known for its adipocyte function. Emerging evidence indicates that PPARγ is also important in anti-inflammatory activity, immune cell regulation, and cell proliferation [4]. PPARγ contains an autonomous transactivation domain 1 (AF-1) in its unstructured N-terminus. The constituted ligand-independent activation of PPARγ target genes occurs through the AF-1 domain. The ligand-binding domain is in the C-terminus, the critical domain for the ligand-dependent transactivation of PPARγ target genes. PPARγ controls gene transcription either through transactivation or transrepression. Many dietary nutrients and phytochemicals modulate PPARγ activity by activating it [30]. However, few studies have also shown an increase in PPARγ protein expression apart from receptor activation [8,31,32]. We carried out PPAR expression studies with 1,8-cineole. 1,8-cineole treatment significantly increased PPARγ protein expression, while the expression of PPARα or PPARβ/δ was not altered. These results indicate that 1,8-cineole specifically increases the expression of the PPARγ transcription factor but not other isoforms. A similar increase in PPARγ protein expression was observed in 1,8-cineole-treated LPS-induced vascular endothelium dysfunction [8]. Next, we evaluated the effect of 1,8 cineole treatment on the NF-κB signaling pathway. NF-κB is an important transcription factor that regulates inflammation [33,34]. The NF-κB subunit P65 was stimulated by DSS administration and this was prevented by 1,8-cineole. These studies indicate that 1,8-cineole exerts anti-inflammatory effects by increasing the expression of PPARγ, which in turn inhibits NF-κB-mediated signaling events [35,36]. The tonic inhibition of COX2 through a long-standing treatment regimen downregulates NF-κB expression in enterocytes [37]. Therefore, it is conceivable that the inhibition of COX2 by 1,8-cineole may also contribute to the inhibition of NF-κB and reduction in inflammation. Apart from COX2 inhibition, other mechanisms may be involved in NF-κB inhibition and reduction in inflammation. Previous studies have shown that the mechanisms involved in suppressing NF-κB and proinflammatory signaling pathways occur through negative gene transcription via the ligand-dependent transrepression process [38,39,40,41].

Dysregulated chemokine production, including the elevation of CXCL1 levels, is seen in the inflamed colonic tissue of IBD patients [42]. To investigate the effect of 1,8-cineole on human colonic cells, we employed HT-29 adenocarcinoma cells challenged with TNF-α to provide an in vitro model of colonic inflammation [43]. 1,8-cineole, even at 200 μM, had minimal effects on HT-29 cell viability at 24 and 48 h. In contrast, 1,8-cineole significantly decreased chemokine IL-8 and CXCL-1 mRNA levels in TNF-α-stimulated HT-29 cells. Furthermore, we evaluated whether 1,8-cineole treatment could alter PPARγ protein expression in HT-29 cells. HT-29 cells express significant levels of PPARγ [44]. 1,8-cineole treatment resulted in the increased expression of PPARγ compared to the TNF-α-stimulated group, but this was similar to the controls. Furthermore, we inhibited PPARγ expression by using siRNA and measured IL-8 and CXCL-1 chemokine mRNA levels in presence of TNF-α. IL-8 chemokine mRNA was significantly lower in the non-silenced PPARγ condition as opposed to silenced. However, no difference in CXCL-1 mRNA expression was observed in the silenced or non-silenced PPARγ conditions. 1,8-cineole treatment also increased PPARγ promoter activity time-dependently. These observations indicate that 1,8-cineole-mediated PPARγ protein expression is at least partly transcription-mediated.

## 4. Materials and Methods

### 4.1. Molecular Docking and Dynamics Studies

A molecular docking study was performed on the PPARγ ligand-binding domain. The crystal structure of the PPARγ ligand-binding domain (PDB ID: 4A4W), resolved at 2.0 Å, was retrieved from a protein databank (www.rcsb.com (accessed on 1 October 2021)). While preparing the protein structure for docking simulation, the bound ligand and water molecules were removed. Furthermore, hydrogen atoms were added and subsequently, the positions of the added hydrogen atoms were optimized with a gradient norm of 0.05 in the Tinker 8 program (Jay Ponder Lab, Department of Chemistry, Washington University, Saint Louis, Missouri 63130 USA, https://dasher.wustl.edu/tinker/ (accessed on 1 October 2021)). The 3D structures of 1,8-cineole and bound ligand amorfrutin B were downloaded from PubChem (https://pubchem.ncbi.nlm.nih.gov (accessed on 1 October 2021)). The molecular docking studies were performed with AutodockVina [45]. To capture the binding site adaptability of the PPARγ binding site, the grid box was set around the center of the bound ligand with the dimensions of 18 × 18 × 18 Å, large enough to search the possible conformational space of the binding site. Molecular docking was performed with an exhaustiveness of 100 to access the entire search space of the binding site. The binding free energy estimates, binding poses, and key interactions were analyzed from the docking results.

The docked complexes of 1,8-cineole with PPARγ and amorfrutin B with PPARγ were subjected to 200 ns molecular dynamics (MD) simulations using the Gromacs 2020.4 program package on an HPC cluster at the Bioinformatics Resources and Applications Facility (BRAF), C-DAC, Pune, Maharastra, India [46,47]. The protein topology was obtained using a CHARMM-36 force field, while the ligand topologies were generated using the CHARMM General Force Field implemented on the CGenFF server. Both systems were solvated with TIP3P water models in a dodecahedron unit cell. The charge on the system was neutralized with the addition of 5 Na^+^ counterions, respectively. The neutralized systems were energy minimized with the steepest descent criteria until the threshold (Fmax < 10 kJ/mol) was reached. Subsequently, the systems were equilibrated at constant volume and temperature conditions (NVT) using a modified Berendsen thermostat at 1 atm pressure and 300 K temperature, respectively, and then at a constant volume and pressure (NPT) using the Berendsen barostat for 1 ns each. The production phase unrestrained 200 ns MD simulations were performed at constant temperature and pressure conditions using the modified Berendsen thermostat and a Parrinello-Rahman barostat [47]. During the production phase MD simulations, covalent bonds were restrained with the LINCS algorithm particle mesh Ewald (PME) method with a cut-off of 1.2 nm employed to measure the long-range electrostatic energies. The resultant trajectories for each system were analysed for the root mean square deviations (RMSD) in the backbone atoms and ligand atoms and the root mean square fluctuations (RMSF) in the residue side-chain atoms, and the number and frequency of hydrogen bonds.

### 4.2. Chemicals, Reagents, and Cells

1,8-cineole was purchased from Santa Cruz Biotechnology and dextran sulfate sodium (DSS) (MW 36,000–50,000 kDa) was procured from MP Biomedicals (Solon, OH, USA). Hexadecyltrimethylammonium bromide (HTAB) and ortho-dianisidine dihydrochloride (ODD) were purchased from Sigma-Aldrich (St. Louis, MO, USA). IL-6, IL-1β, TNF-α, and IL-17A ELISA kits were purchased from R&D systems (Minneapolis, MN, USA). The reverse transcription kit was procured from Applied Biosystems (Foster City, CA, USA). EvaGreen 5× Master Mix from Solis BioDyne (Tartu, Estonia) and Macrogen Inc. (Seoul, South Korea) provided the primers for quantitative RT-PCR. The protease and phosphatase inhibitors were supplied by Thermo Fisher Scientific (Rockford, IL, USA). Antibodies were purchased from Santacruz Biotechnology (Dallas, TX, USA) and Thermo Fisher Scientific (Rockford, IL, USA). Catalogue numbers were given in our previous publication [31]. Commercially available pNL 1.3 and pNL1.3.CMV vectors were purchased from Promega (Cat# N1021 and N1101; Madison, WI, USA) and the PPRE-pNL1.3 plasmid was purchased from Addgene (Cat#84394; Watertown, MA, USA). Other reagents were obtained from the suppliers listed in our previous publication [31]. The HT-29 colorectal adenocarcinoma cells were obtained from the American Type Culture Collection (Manassas, VA, USA).

### 4.3. Animals

C57BL/6J mice (12 weeks old) weighing 25–30 g were procured from the Central Animal Facility, CMHS, UAE University. Two animals per cage were kept one week prior to the commencement of each experiment for acclimatization. The animals were kept at a temperature of 23 ± 1 °C, a 12 h light and dark cycle, and 50–60% humidity. Food and water were provided ad libitum. The UAEU Institutional Animal Ethical Committee approved the present study (approval # ERA_2020_6079, approval date 14 May 2020).

### 4.4. Experimental Design

Mice were randomly allocated to 5 groups (with 8 animals in each group). Group I: Untreated control. Group II: DSS alone. Group III: DSS + Cineole (CIN) (100 mg/kg body weight/day). Group IV: DSS + CIN (200 mg/kg body weight/day). Group V: DSS + SAZ (50 mg/kg body weight/day). DSS (2%) was freshly prepared every day in autoclaved drinking water. At the end of the 8-day treatment protocol, the animals were euthanized using a pentobarbital overdose (100 mg/kg body weight). Colons were surgically removed and length measured for DAI, including the caecum. Colons were then cleaned with ice-cold saline to remove fecal content. After cleaning, the colon was scraped to separate the mucus layer and immediately snap-frozen using liquid nitrogen and stored at −80 °C until further use. Colon pieces 5 mm in length were cut and then fixed using 10% formalin for hematoxylin and eosin (H&E) staining.

### 4.5. Evaluation of Disease Activity Index (DAI) Score

The mice were weighed daily and observed for the presence of loose stools, diarrhea, and bleeding. The DAI scores were calculated based on parameters as published in our previous study [48]. In short, the DAI scores comprise the scores acquired from the daily recordings of loss of weight, loose stools/diarrhea, and bleeding.

### 4.6. Proinflammatory Cytokine Measurement by Enzyme-linked Immunosorbent Assay (ELISA)

Colonic mucosa was homogenized with a phosphatase and protease inhibitor cocktail tablet (Cat#A32959; Thermo Fisher Scientific, Rockford, IL, USA) dissolved in a RIPA buffer (Cat#20-188; Millipore, St. Louis, MO, USA) with zirconium beads (2 mm, Cat#11079124zx; Biospec, Bartlesville, OK, USA) in a Precellys 24-tissue homogenizer (Bertin Instruments, Montigny-le-Bretonneux, France). The resultant homogenate was centrifuged at 1000 × *g* at 4 °C. After centrifugation, the supernatant was transferred into a fresh microcentrifuge tube gently mixing on the tube rotator overnight at 4 °C. The homogenate was centrifuged at 15,000 × *g* at 4 °C for 30 min, diluted (1:3) with RIPA buffer, and its protein concentration was estimated using a Pierce BCA Protein Assay Kit (Cat#23225; Thermo Fisher Scientific, Rockford, IL, USA). Undiluted homogenate was kept in aliquots at −80 °C for later use. TNF-α, IL-1β, IL-6, and IL-17A cytokines were determined by ELISA assay in the colonic mucosal homogenates according to the manufacturer’s instructions.

### 4.7. Myeloperoxidase (MPO) Assay

Tissue MPO activity was measured as described previously [49]. Briefly, 25 mg of freshly scraped colonic mucosa was homogenized using zirconium beads in 50 mM phosphate buffer (pH 6) containing 0.5% hexadecyltrimethylammonium bromide (HTAB). Homogenates were processed through a freeze–thaw cycle (liquid nitrogen and a 25 °C water bath) and sonicated for 30 s. This process was repeated three times. Suspensions were then centrifuged at 20,000× *g* for 20 min at 4 °C. The supernatant (0.1 mL) was flooded with 2.9 mL of 50 mM phosphate buffer (pH 6) containing 0.53 mM of o-dianisidine hydrochloride and 0.15 mM hydrogen peroxide. The change in absorbance was measured every 15 s for 5 min at 460 nm. These results were expressed in units (U) of MPO/mg of protein. The protein concentration of supernatants was determined by a Pierce BCA Protein Assay Kit (Pierce, Rockford, IL, USA). MPO activity was determined as the mean absorbance at 460 nm/incubation time/protein concentration.

### 4.8. Histopathological Evaluation

The colon was fixed using a 10% formaldehyde solution overnight. The dehydration process was carried out using ethanol in increasing order of concentration. The dehydrated tissues were embedded in paraffin. The paraffin-embedded colon was cut into thin slices (2 μm thick) and stained using H&E for histological analysis. To evaluate the changes in histopathology, a clinical pathologist determined each sample’s score. These samples were blinded for histopathological evaluation. Inflammation score = Grade of inflammation × Percentage of involvement. The colonic inflammation degree was determined as per a previously published study [50].

### 4.9. RNA Extraction and Real-Time RT-PCR

RNA extraction from the colonic mucosa and conversion into cDNA and real-time PCR were performed as described previously [32]. The 18s gene product was used as an internal reference gene in the present study. The change in CT values was calculated using the delta CT method (2^−ΔΔCT^) [51]. Primer sequences for all genes used in the present study were reported in our previous study [52]. The primers used for the real-time PCR analysis were as follows (Table 2).

### 4.10. Western Blot

The frozen samples of colonic mucosa were homogenized in a RIPA buffer with a protease and phosphatase inhibitor cocktail using a bead homogenizer as previously described [52]. The undiluted homogenate protein concentrations were determined using a Pierce BCA Protein Assay Kit (Cat#23225; Thermo Fisher Scientific, Rockford, IL, USA). A total of 20 µg (colonic tissue and HT-29 cells) of proteins was resolved using sodium dodecyl sulfate-polyacrylamide gel electrophoresis (SDS-PAGE) using 8–12% gels and subsequently transferred onto the PVDF membrane. These PVDF membranes were immune-blotted using specific antibodies COX-2, iNOS, PPAR-α, PPAR-β/δ, PPARγ, p^Ser536^ NFκB p65, HO-1, NQO1, Nrf2, and Keap-1 The internal controls used to normalize the blots were GAPDH and Lamin-B. Western blot bands were densitometrically examined using the free software ImageJ (Version 1.51j8, https://imagej.nih.gov/ij/download.html, accessed on 17 November 2022). In order to perform optical density (OD) calculations, images were captured by an Azure Sapphire™ (Dublin, OH, USA) biomolecular imager and transformed into 8-bit format. Each band was chosen individually, circumscribed using the rectangular ROI selection, and the peak areas were then measured. Figure 1 displays the bands’ measured pixel intensities by Image J.

### 4.11. Measurement of Superoxide Dismutase, Catalase Enzyme Activity, and Tissue Nitrite Concentration

Superoxide dismutase (SOD) was assayed using the method by Kakkar et al. [53] based on a 50% inhibition of the formation of NADH–phenazine methosulphate–nitro blue tetrazolium (NBT) formazan at 520 nm. One unit of the enzyme was taken as the amount of enzyme required for the 50% inhibition of NBT reduction/min/mg protein. Catalase activity (CAT) was determined using the method by Sinha [54]. The values of CAT activity were expressed as moles of H_2_O_2_ utilized/min/mg protein. The levels of nitric oxide (NO) in the colon homogenate were measured using a Griess reagent using the method by Lu et al. [55]; nitrite concentration, an indicator of NO production, was calculated from a NaNO_2_ standard curve and expressed as µmol/mg protein. Snap-frozen colon samples were processed for biochemical estimation on the following day of sample collection.

### 4.12. HT-29 Cell Culture

HT-29 colon cancer cells were cultured at 37 °C and 5% CO_2_ in a humidified incubator in high-glucose DMEM, containing 100 U/mL penicillin, 100 µg/mL streptomycin, and 10% (*v/v*) heat-inactivated fetal bovine serum (FBS). Cultured cells were seeded (1.5 × 10^5^ cells per well) onto 6-well plates 24 h before treatment. To induce inflammation, the HT-29 cells were treated with 1 ng/mL TNF-α with or without 1,8-cineole (20 and 40 μM, 1,8-cineole MW 154.249 g/mol) for 24 h. After treatment, the medium was collected for the expression of chemokines (IL-8 and CXCL-1) and the cells were collected with a RIPA buffer containing protease inhibitors for the measurement of PPARγ, protein, and mRNA. HT-29 cells were used for PPARγ siRNA knockdown and the promotor/nanoluciferase assay.

### 4.13. Cell Viability Assay

HT-29 cells (5000 cells/well) were seeded onto 96-well plates and treated with a range of cineole concentrations (0, 12.5, 50, 100, and 200 µM) for 24 and 48 h. According to the manufacturer’s instructions, cell viability was determined using the CellTiter-Glo^®^ Cell Viability Kit (Cat#G7571, Promega, Madison, WI, USA) after the indicated treatment period. Luminescence was measured using a Tecan Infinite^®^ 200 PRO (Männedorf, Switzerland) plate reader. Data were represented as the percentage of viable cells (quantified by ATP content) in the 1,8-cineole-treated groups compared to the untreated, control group.

### 4.14. PPARγ Promotor and Nanoluciferase Assay

Vectors and plasmids (expressing luminescent NanoLuc^®^ luciferase, Cat#N1120, Promega, Madison, WI, USA) were transformed into JM109-competent bacterial cells by a ligation method and cultured overnight in a 125 mL medium at 37 °C. The plasmid was extracted using the Plasmid Purification Maxiprep Kit (Cat#12162; Qiagen, Germany) as per the manufacturers’ instructions. The plasmid DNA and lipofectamine LTX mixture were prepared in a serum and antibiotic-free DMEM medium as described previously [31]. The transient transfection was performed with PPRE-pNL1.3 or pNL1.3 and basic secreted luciferase reporter used as the control. HT-29 cells were transfected in 24-well plates (Cat#3516; Corning Costar, Kennebunk, ME, USA) using Lipofectamine LTX with Plus Reagent (Invitrogen, Carlsbad, CA, USA) following the manufacturer’s protocol. After 24 h, the Lipofectamine LTX added medium was removed and replaced with fresh 2% FBS medium containing cineole at 40 μM, with 10 μM GW 1929 (PPARγ agonist) and GW9662 (PPARγ antagonist). After 24 and 48 h of treatment, 20 μL of each cell supernatant was dispensed into a 96-well black plate (Cat#237105; Thermo Fisher Scientific, Rockford, IL, USA) and the secreted NanoLuc luciferase activity was determined using Nano-Glo^®^ Luciferase Assay buffer (Cat#N1120; Promega, Madison, WI, USA) according to the manufacturer’s instructions. Luminescence in each well was then measured by using a Tecan multimode (Infinite 200 PRO, Männedorf, Switzerland) plate reader.

### 4.15. PPARγ Knockdown Mediated by siRNA

Using the Lipofectamine LTX Plus Transfection Kit (Invitrogen, Carlsbad, CA, USA), HT-29 cells were seeded into 24-well plates (40,000 cells/well) and transfected with 30 nM PPAR Silencer Select siRNA (ID s10888) or 30 nM Silencer Select Negative Control 1 siRNA (Ambion, Life Technologies; Carlsbad, CA, USA) for 2 days. After 48 h of transfection, the medium was removed and cells were then treated with 1,8-cineole 40 µM or the vehicle control in growth medium for an additional 24 h prior to RNA/protein collection. Protein/RNA were extracted and a Western blot analysis was used to confirm the expression knockdown.

### 4.16. Statistical Analysis

Statistical analysis was performed using GraphPad Prism (version 9.0) software (San Diego, CA, USA). Most of the group data were compared by analysis of variance (ANOVA). For multiple comparisons, Tukey’s post hoc test was used. The PPARγ siRNA-treated mRNA expression data were analyzed using Student’s *t*-test between the control and siRNA-treated groups. Data are represented as the means ± S.E.M and a *p* value < 0.05 was considered to be statistically significant.

## 5. Conclusions

Our results indicate that 1,8-cineole is a PPARγ agonist that mediates anti-inflammatory action by stimulating colonic PPARγ protein expression and suppressing the NF-κB signaling pathway. 1,8-cineole activates the Nrf2/Keap1 system to exert potent antioxidant effects by enhancing HO1, NQO1 protein, and mRNA expression and by decreasing SOD and CAT enzyme activity. 1,8-cineole also increased PPARγ promoter activity, indicating that the observed increase in PPARγ protein expression is, at least in part, transcriptionally regulated.

## Figures and Tables

**Table 1:** Molecular docking results.

Compound	Binding Free Energy Estimate (kcal/mol)	Interactions	
		H-Bond Interactions	Hydrophobic Interactions
1,8-Cineole	−5.7	-	Ser342, Cys285, Ile341, Leu340, Leu333, Met363, Ser289, Arg288, Val339, and Leu330
Amorfrutin B	−6.9	Ser342	Ser342, Ile341, Gly284, Leu255, Ile281, Arg288, Met329, Leu330, Ile326, Ala292, Leu333, Met348, Leu353, Val339, Met364, and Cys285

**Table 2:** Primer sequences used for real-time PCR.

Gene	Forward	Reverse	PMID
*Mouse IL-6*	5′-TGTGTCGTGCTGTTCAGAACC-3′	5′-AGGAATCCCGCAATGATGG-3′	22326488
*Mouse Il-1β*	5′-TCGCTCAGGGTCACAAGAAA-3′	5′-CATCAGAGGCAAGGAGGAAAC-3′	21735552
*Mouse TNF-α*	5′-AGGCTGCCCCGACTACGT-3′	5′-GACTTTCTCCTGGTATGAGATAGCAAA-3′	21705622
*Mouse IL-17A*	5′-ATCCCTCAAAGCTCAGCGTGTC-3′	5′-GGGTCTTCATTGCGGTGGAGAG-3′	18606690
*Mouse COX2*	5′-AACCGCATTGCCTCTGAAT-3′	5′-CATGTTCCAGGAGGATGGAG-3′	22158945
*Mouse iNOS*	5′-CGAAACGCTTCACTTCCAA-3′	5′-TGAGCCTATATTGCTGTGGCT-3′	22158945
*Mouse 18S*	5′-CCCCTCGATGACTTTAGCTGAGTGT-3′	5′-CGCCGGTCCAAGAATTTCACCTCT-3′	22427817
*Human CXCL-1*	5′-GCGGAAAGCTTGCCTCAATC-3′	5′-GGTCAGTTGGATTTGTCACTGT-3′	25938459
*Human IL-8*	5′-ACTGAGAGTGATTGAGAGTGGAC-3′	5′-AACCCTCTGCACCCAGTTTTC-3′	31273598
*Human PPARγ*	5′-TTCAAGAGTACCAAAGTGCAATCAA-3′	5′-AATAAGGTGGAGATGCAGGCTC-3′	20421464
*Human GAPDH*	5′-TCAAGGCTGAGAACGGGAAG-3′	5′-CGCCCCACTTGATTTTGGAG-3′	33575432

## Data Availability

Data are available upon request from the corresponding author.

## Supplementary Materials

The following supporting information can be downloaded at: https://www.mdpi.com/article/10.3390/ijms24076160/s1.
