# Supplementary material for: Molecular Docking Identifies 1,8-Cineole (Eucalyptol) as A Novel PPARγ Agonist That Alleviates Colon Inflammation
Source: Int J Mol Sci. 2023 Mar 24;24(7):6160. doi: 10.3390/ijms24076160 (PMC10094723; doi:10.3390/ijms24076160)
Supplement: Supplementary file 1 [file ijms-24-06160-s001.zip › ijms-2253672-supplementary.pdf]

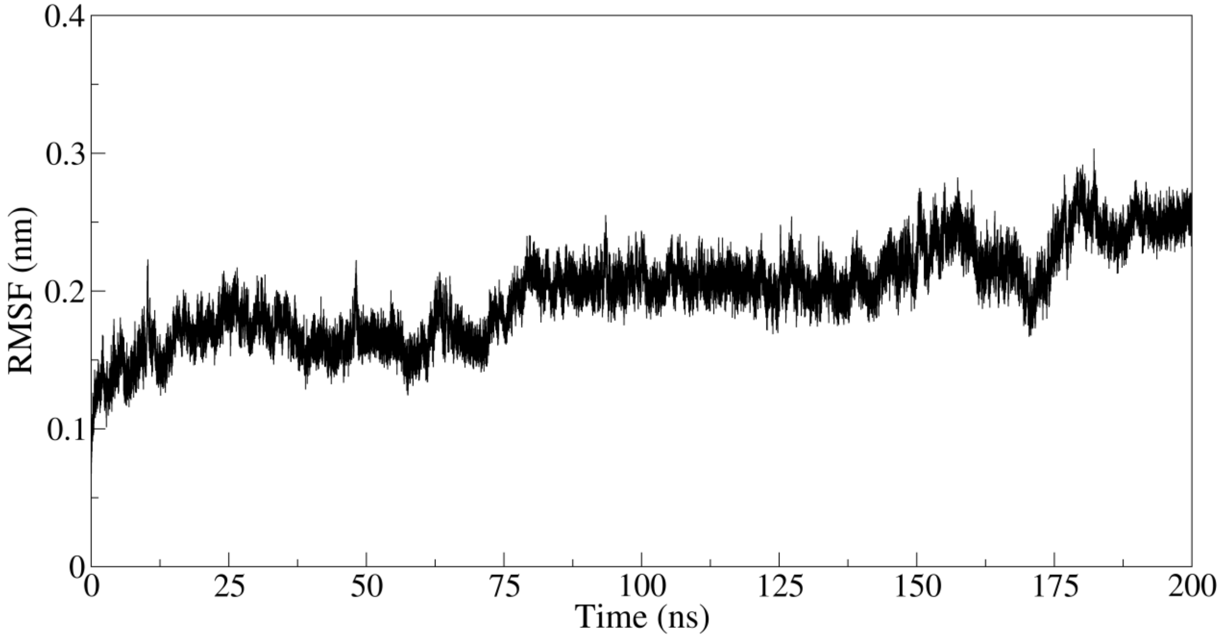

Figure S1. RMSD in apo-PPAR-γ backbone atoms

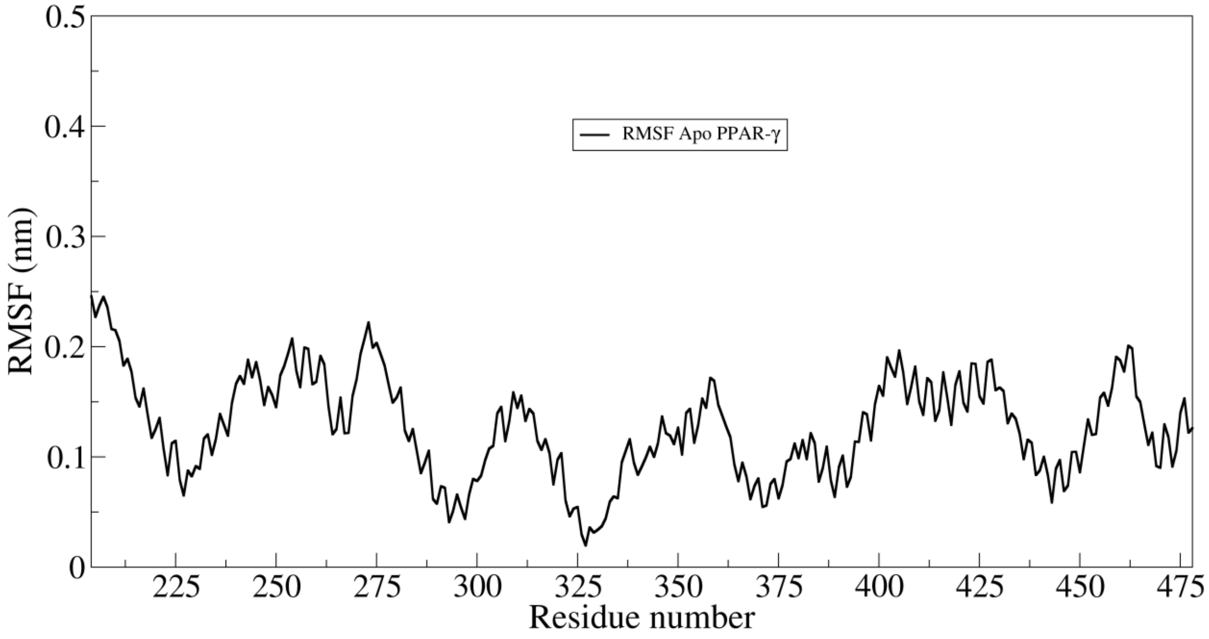

Figure S2. RMSF in apo-PPAR-γ residues

RAW Data Sheet:

Disease activity index:

	day1	day2	day3	day4	day5	day6	day7	day8	
Control1	0	0	0	0	0	0	0	0	0
Control2	0	0	0	0	0	0	0	0	0
Control3	0	0	0	0	0	0	0	0	0
Control4	0	0	0	0	0	0	0	0	0
Control5	0	0	0	0	0	0	0	0	0
Control6	0	0	0	0	0	0	0	0	0
Control7	0	0	0	0	0	0	0	0	0
Control8	0	0	0	0	0	0	0	0	0
**Avg=**	**0.00**	**0.00**	**0.00**	**0.00**	**0.00**	**0.00**	**0.00**	**0.00**	
**SD=**	**0.00**	**0.00**	**0.00**	**0.00**	**0.00**	**0.00**	**0.00**	**0.00**	
**SEM=**	**0.00**	**0.00**	**0.00**	**0.00**	**0.00**	**0.00**	**0.00**	**0.00**	
DSS1	0	0	0	0	1	1	2	3	0.875
DSS2	0	0	1	2	2	2	3	3	1.625
DSS3	0	1	1	1	2	3	3	3	1.75
DSS4	0	0	0	1	2	2	2	3	1.25
DSS5	0	0	1	1	2	2	3	4	0.00
DSS6	0	0	0	1	2	2	3	4	1.5
DSS7	0	0	1	1	2	2	2	3	1.375
DSS8	0	0	0	1	1	2	2	3	1.125
**Avg=**	**0.00**	**0.13**	**0.50**	**1.00**	**1.75**	**2.00**	**2.50**	**3.25**	
**SD=**	**0.00**	**0.35**	**0.53**	**0.53**	**0.46**	**0.53**	**0.53**	**0.46**	
**SEM=**	**0.00**	**0.13**	**0.19**	**0.19**	**0.16**	**0.19**	**0.19**	**0.16**	
DSS-Cin-1	0	0	0	1	1	2	2	2	1
DSS-Cin-1	0	0	0	1	1	2	2	2	1
DSS-Cin-1	0	0	0	1	1	2	2	2	1
DSS-Cin-1	0	0	0	1	1	1	2	2	0.875
DSS-Cin-1	0	0	0	1	2	2	3	3	1.375
DSS-Cin-1	0	0	1	1	1	1	2	2	1
DSS-Cin-1	0	0	0	1	1	2	2	2	1
DSS-Cin-1	0	0	1	1	1	2	2	2	1.125
**Avg=**	**0.00**	**0.00**	**0.25**	**1.00**	**1.13**	**1.75**	**2.13**	**2.13**	
**SD=**	**0.00**	**0.00**	**0.46**	**0.00**	**0.35**	**0.46**	**0.35**	**0.35**	
**SEM=**	**0.00**	**0.00**	**0.16**	**0.00**	**0.13**	**0.16**	**0.13**	**0.13**	

DSS-Cin-200-1	0	0	0	0	1	1	1	2	0.625
DSS-Cin-200-2	0	0	0	0	1	1	1	1	0.5
DSS-Cin-200-3	0	0	0	0	1	1	1	1	0.5
DSS-Cin-200-4	0	0	0	0	1	1	2	2	0.75
DSS-Cin-200-5	0	0	0	1	1	1	1	2	0.75
DSS-Cin-200-6	0	0	0	0	1	1	1	1	0.5
DSS-Cin-200-7	0	0	0	0	1	1	1	1	0.5
DSS-Cin-200-8	0	0	0	0	1	1	1	2	0.625
**Avg=**	**0.00**	**0.00**	**0.00**	**0.13**	**1.00**	**1.00**	**1.13**	**1.50**	
**SD=**	**0.00**	**0.00**	**0.00**	**0.35**	**0.00**	**0.00**	**0.35**	**0.53**	
**SEM=**	**0.00**	**0.00**	**0.00**	**0.13**	**0.00**	**0.00**	**0.13**	**0.19**	
DSS-SAZ-1	0	0	0	1	1	2	2	2	1
DSS-SAZ-2	0	0	1	1	1	2	2	3	1.25
DSS-SAZ-3	0	0	1	1	1	1	2	2	1
DSS-SAZ-4	0	0	0	1	1	1	2	2	0.875
DSS-SAZ-5	0	0	0	1	1	2	2	2	1
DSS-SAZ-6	0	0	0	0	1	1	2	2	0.75
DSS-SAZ-7	0	0	0	0	1	1	2	3	0.875
DSS-SAZ-8	0	0	1	1	1	1	1	2	0.875
**Avg=**	**0.00**	**0.00**	**0.38**	**0.75**	**1.00**	**1.38**	**1.88**	**2.25**	
**SD=**	**0.00**	**0.00**	**0.52**	**0.46**	**0.00**	**0.52**	**0.35**	**0.46**	
**SEM=**	**0.00**	**0.00**	**0.18**	**0.16**	**0.00**	**0.18**	**0.13**	**0.16**	

	**Day-1**	**Day-2**	**Day-3**	**Day-4**	**Day-5**	**Day-6**	**Day-7**	**Day-8**
Con	0	0	0	0	0	0	0	0
DSS	0	0.125	0.5	1	1.75	2	2.5	3.25
DSS-cin-100	**0.00**	**0.00**	**0.25**	**1.00**	**1.13**	**1.75**	**2.13**	**2.13**
DSS-Cin-200	0	0	0	0.125	1	1	1.125	1.5
DSS-SAZ	0	0	0.375	0.75	1	1.375	1.875	2.25

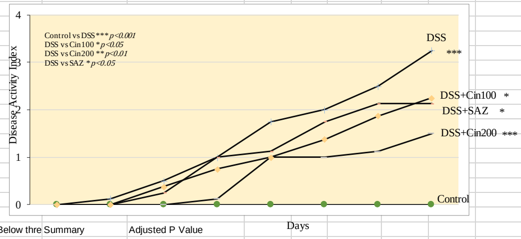

Table Anal	Data 63			
Data sets	A-E			
ANOVA summary				
F	28.14			
P value	<0.0001			
P value su	****			
Significant	Yes			
R squared	0.2632			
Brown-Forsythe test				
F (DFn, DF	31.37 (4, 315)			
P value	<0.0001			
P value su	****			
Are SDs si	Yes			
Bartlett's test				
Bartlett's statistic (corrected)				
P value				
P value summary				
Are SDs significantly different (P < 0.05)?				
ANOVA ta	SS	DF	MS	F (DFn, DFd)
Treatment	80.61	4	20.15	F (4, 315) = 28.14
Residual (v	225.6	315	0.7162	
Total	306.2	319		
Data summary				
Number of	5			
Number of	320			

Number of families	1
Number of comparisons p	10
Alpha	0.05

Tukey's multiple comparis	Mean Diff.	95.00% CI	Below thre	Summary	Adjusted P Value	
Control vs. DSS	-1.422	-1.800 to -	Yes	****	<0.0001	A-B
Control vs. DSS-Cin100	-0.9844	-1.363 to -	Yes	****	<0.0001	A-C
Control vs. DSS-Cin200	-0.5313	-0.9094 to	Yes	**	0.0013	A-D
Control vs. DSS-SAZ	-1.016	-1.394 to -	Yes	****	<0.0001	A-E
DSS vs. DSS-Cin100	0.4375	0.05932 to	Yes	*	0.0142	B-C
DSS vs. DSS-Cin200	0.8906	0.5124 to 1	Yes	****	<0.0001	B-D
DSS vs. DSS-SAZ	0.4063	0.02807 to	Yes	*	0.0282	B-E
DSS-Cin100 vs. DSS-Cin	0.4531	0.07494 to	Yes	**	0.0099	C-D
DSS-Cin100 vs. DSS-SA	-0.03125	-0.4094 to	No	ns	0.9994	C-E
DSS-Cin200 vs. DSS-SA	-0.4844	-0.8626 to	Yes	**	0.0046	D-E

Test details	Mean 1	Mean 2	Mean Diff.	SE of diff.	n1	n2	q	DF
Control vs. DSS	0	1.422	-1.422	0.1378	64	64	14.59	315
Control vs. DSS-Cin100	0	0.9844	-0.9844	0.1378	64	64	10.1	315
Control vs. DSS-Cin200	0	0.5313	-0.5313	0.1378	64	64	5.451	315
Control vs. DSS-SAZ	0	1.016	-1.016	0.1378	64	64	10.42	315
DSS vs. DSS-Cin100	1.422	0.9844	0.4375	0.1378	64	64	4.489	315
DSS vs. DSS-Cin200	1.422	0.5313	0.8906	0.1378	64	64	9.138	315
DSS vs. DSS-SAZ	1.422	1.016	0.4063	0.1378	64	64	4.168	315
DSS-Cin100 vs. DSS-Cin	0.9844	0.5313	0.4531	0.1378	64	64	4.649	315
DSS-Cin100 vs. DSS-SA	0.9844	1.016	-0.03125	0.1378	64	64	0.3206	315
DSS-Cin200 vs. DSS-SA	0.5313	1.016	-0.4844	0.1378	64	64	4.97	315

Colon length:

Control	DSS	DSS+Cin100	DSS+Cin200	DSS+SAZ
9.5	4.5	6.5	9	6
10	5.5	7	7.5	6.5
8.5	5	7.25	8.25	5.5
10	4.25	6.75	7.5	7
9	5.25	5.5	6	5.75
8	6	5	7	6.25
8.5	5.75	7.25	8.5	6.75
9.25	4	6	6	7.5

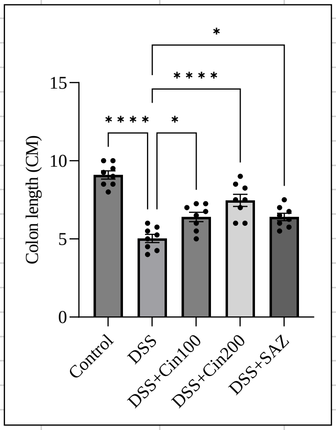

Table Anal	DSS-Cin-Colon length
Data sets	A-E
ANOVA summary	
F	26.63
P value	<0.0001
P value su	****
Significant	Yes
R squared	0.7527
Number of	1
Number of	10
Alpha	0.05

ANOVA ta	SS	DF	MS	F (DFn, DF	P value
Treatment	72.91	4	18.23	F (4, 35) =	P<0.0001
Residual (\	23.96	35	0.6846		
Total	96.87	39			
Data summary					
Number of	5				
Number of	40				

Tukey's m	Mean Diff.	95.00% CI of d	Below threshold	Summary	Adjusted P Value	
Control vs.	4.063	2.873 to 5.252	Yes	****	<0.0001	A-B
Control vs.	2.688	1.498 to 3.877	Yes	****	<0.0001	A-C
Control vs.	1.625	0.4356 to 2.81	Yes	**	0.0033	A-D
Control vs.	2.688	1.498 to 3.877	Yes	****	<0.0001	A-E
DSS vs. D	-1.375	-2.564 to -0.18	Yes	*	0.0167	B-C
DSS vs. D	-2.438	-3.627 to -1.24	Yes	****	<0.0001	B-D
DSS vs. D	-1.375	-2.564 to -0.18	Yes	*	0.0167	B-E
DSS+Cin1	-1.063	-2.252 to 0.126	No	ns	0.0987	C-D
DSS+Cin1	0	-1.189 to 1.189	No	ns	>0.9999	C-E
DSS+Cin2	1.063	-0.1269 to 2.25	No	ns	0.0987	D-E

Test detail	Mean 1	Mean 2	Mean Diff.	SE of diff.	n1	n2	q	DF
Control vs.	9.094	5.031	4.063	0.4137	8	8	13.89	35
Control vs.	9.094	6.406	2.688	0.4137	8	8	9.187	35
Control vs.	9.094	7.469	1.625	0.4137	8	8	5.555	35
Control vs.	9.094	6.406	2.688	0.4137	8	8	9.187	35
DSS vs. D	5.031	6.406	-1.375	0.4137	8	8	4.7	35
DSS vs. D	5.031	7.469	-2.438	0.4137	8	8	8.332	35
DSS vs. D	5.031	6.406	-1.375	0.4137	8	8	4.7	35
DSS+Cin1	6.406	7.469	-1.063	0.4137	8	8	3.632	35
DSS+Cin1	6.406	6.406	0	0.4137	8	8	0	35
DSS+Cin2	7.469	6.406	1.063	0.4137	8	8	3.632	35

MPO activity:

Control	DSS	DSS+Cin100	DSS+Cin200	DSS+SAZ
0.18099	0.6875361	0.409246878	0.269060634	0.440078567
0.217134	0.7751654	0.505105028	0.281463098	0.320335986
0.145844	0.40128	0.318879346	0.271425951	0.31045862
0.181323	0.6213272	0.544410417	0.273983228	0.356957724
0.084242	0.5548352	0.115381017	0.124235949	0.143784573
0.15223	0.611789	0.322581	0.1955623	0.2988452
0.175996	0.4778923	0.2899652	0.2447893	0.3614789
0.200459	0.5422589	0.78125	0.174122	0.29665478

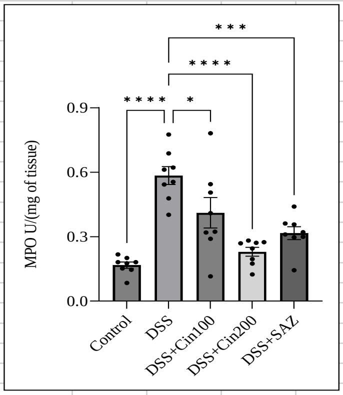

Table Anal	DSS-Cin-MPO activity
Data sets	A-E
ANOVA summary	
F	16.19
P value	<0.0001
P value su	****
Significant	Yes
R squared	0.6492

ANOVA ta	SS	DF	MS	F (DFn, DFd)	P value
Treatment	0.8576	4	0.2144	F (4, 35) = 16.1	P<0.0001
Residual (v	0.4634	35	0.01324		
Total	1.321	39			

Data summary	
Number of	5
Number of	40
Number of	1
Number of	10
Alpha	0.05

Tukey's mu	Mean Diff.	95.00% CI of diff.	Below threshold?	Summary	Adjusted P	Value
Control vs.	-0.4167	-0.5821 to -0.2513	Yes	****	<0.0001	A-B
Control vs.	-0.2436	-0.4090 to -0.0781	Yes	**	0.0014	A-C
Control vs.	-0.06205	-0.2275 to 0.1034	No	ns	0.8162	A-D
Control vs.	-0.1488	-0.3142 to 0.0166	No	ns	0.095	A-E
DSS vs. D	0.1732	0.007742 to 0.338	Yes	*	0.0364	B-C
DSS vs. D	0.3547	0.1893 to 0.5201	Yes	****	<0.0001	B-D
DSS vs. D	0.2679	0.1025 to 0.4334	Yes	***	0.0004	B-E
DSS+Cin1	0.1815	0.01611 to 0.3469	Yes	*	0.0256	C-D
DSS+Cin1	0.09478	-0.07064 to 0.260	No	ns	0.4785	C-E
DSS+Cin2	-0.08674	-0.2522 to 0.0786	No	ns	0.5645	D-E

Test detail	Mean 1	Mean 2	Mean Diff.	SE of diff.	n1	n2	q	DF
Control vs.	0.1673	0.584	-0.4167	0.05753	8	8	10.24	35
Control vs.	0.1673	0.4109	-0.2436	0.05753	8	8	5.987	35
Control vs.	0.1673	0.2293	-0.06205	0.05753	8	8	1.525	35
Control vs.	0.1673	0.3161	-0.1488	0.05753	8	8	3.657	35
DSS vs. D	0.584	0.4109	0.1732	0.05753	8	8	4.256	35
DSS vs. D	0.584	0.2293	0.3547	0.05753	8	8	8.718	35
DSS vs. D	0.584	0.3161	0.2679	0.05753	8	8	6.586	35
DSS+Cin1	0.4109	0.2293	0.1815	0.05753	8	8	4.462	35
DSS+Cin1	0.4109	0.3161	0.09478	0.05753	8	8	2.33	35
DSS+Cin2	0.2293	0.3161	-0.08674	0.05753	8	8	2.132	35

Histology inflammation score:

Control	DSS	DSS-Cin100	DSS-Cin200	DSS-SAZ
0	8	8	5	10
0	10	6	5	6
0	12	9	4	7
0	8	6	4	8
0	9	5	4	4
0	10	7	4	5
0	11	5	4	6
0	12	10	4	4
0	7	4	5	5
0	8	6	4	6
0	6	7	5	5
0	10	8	3	4
0	10	4	4	7
0	11	5	3	8
0	12	6	3	5
0	10	6	4	4
0	9	7	4	5
0	7	5	5	6
0	8	7	5	5
0	6	8	3	5
0	9	6	4	6
0	10	7	6	5
0	11	6	3	6
0	12	5	5	7

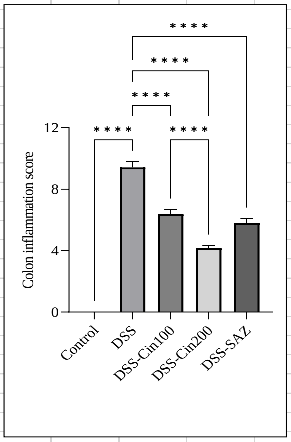

Table Anal	Histology-Colon inflammation score							
Data sets	A-E							
ANOVA summary								
F	167							
P value	<0.0001							
P value su	****							
Significant	Yes							
R squared	0.8531							
ANOVA ta	SS	DF	MS	F (DFn, DFd)	P value			
Treatment	1143	4	285.6	F (4, 115) = 1	P<0.0001			
Residual (v	196.8	115	1.711					
Total	1339	119						
Data summary								
Number of	5							
Number of	120							
Number of	1							
Number of	10							
Alpha	0.05							
Tukey's m	Mean Diff.	95.00% CI of diff.	Below threshold'	Summary	Adjusted P	Value		
Control vs.	-9.417	-10.46 to -8.370	Yes	****	<0.0001	A-B		
Control vs.	-6.375	-7.421 to -5.329	Yes	****	<0.0001	A-C		
Control vs.	-4.167	-5.213 to -3.120	Yes	****	<0.0001	A-D		
Control vs.	-5.792	-6.838 to -4.745	Yes	****	<0.0001	A-E		
DSS vs. D	3.042	1.995 to 4.088	Yes	****	<0.0001	B-C		
DSS vs. D	5.25	4.204 to 6.296	Yes	****	<0.0001	B-D		
DSS vs. D	3.625	2.579 to 4.671	Yes	****	<0.0001	B-E		
DSS-Cin10	2.208	1.162 to 3.255	Yes	****	<0.0001	C-D		
DSS-Cin10	0.5833	-0.4632 to 1.630	No	ns	0.5357	C-E		
DSS-Cin20	-1.625	-2.671 to -0.5785	Yes	***	0.0003	D-E		
Test detail	Mean 1	Mean 2	Mean Diff.	SE of diff.	n1	n2	q	DF
Control vs.	0	9.417	-9.417	0.3776	24	24	35.27	115
Control vs.	0	6.375	-6.375	0.3776	24	24	23.88	115
Control vs.	0	4.167	-4.167	0.3776	24	24	15.61	115
Control vs.	0	5.792	-5.792	0.3776	24	24	21.69	115
DSS vs. D	9.417	6.375	3.042	0.3776	24	24	11.39	115
DSS vs. D	9.417	4.167	5.25	0.3776	24	24	19.66	115
DSS vs. D	9.417	5.792	3.625	0.3776	24	24	13.58	115
DSS-Cin10	6.375	4.167	2.208	0.3776	24	24	8.271	115
DSS-Cin10	6.375	5.792	0.5833	0.3776	24	24	2.185	115
DSS-Cin20	4.167	5.792	-1.625	0.3776	24	24	6.086	115

DSS-Cin-IL6-ELISA:

Control	DSS	DSS-Cin100	DSS-Cin200	DSS-SAZ
25.38312	151.0334	36.61762	31.70833	37.1001
33.89248	92.93752	42.48178	47.27477	36.35765
27.53361	117.7063	67.44186	42.72671	43.51486
37.84943	142.4505	59.17841	22.97464	55.73486
14.50358	78.00801	75.62007	19.87972	53.37971
16.62886	108.9013	66.9317	28.97226	43.32065
11.12404	119.0233	49.3776	48.91726	62.4056
12.33457	104.9249	47.62304	19.45899	46.82283

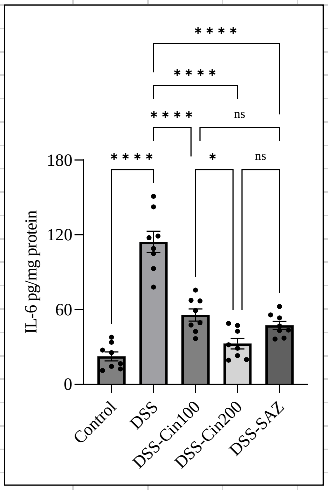

Table Anal	DSS-Cin-IL6-ELISA
Data sets	A-E
ANOVA summary	
F	46.56
P value	<0.0001
P value su	****
Significant	Yes
R squared	0.8418

ANOVA ta	SS	DF	MS	F (DFn, DF	P value
Treatment	41129	4	10282	F (4, 35) =	P<0.0001
Residual (\	7729	35	220.8		
Total	48858	39			

Data summary	
Number of	5
Number of	40
Number of	1
Number of	10
Alpha	0.05

Tukey's m	Mean Diff.	95.00% CI of diff	Below threshold?	Summary	Adjusted P	Value
Control vs.	-91.97	-113.3 to -70.61	Yes	****	<0.0001	A-B
Control vs.	-33.25	-54.61 to -11.89	Yes	***	0.0007	A-C
Control vs.	-10.33	-31.69 to 11.03	No	ns	0.6375	A-D
Control vs.	-24.92	-46.29 to -3.561	Yes	*	0.0155	A-E
DSS vs. D	58.71	37.35 to 80.08	Yes	****	<0.0001	B-C
DSS vs. D	81.63	60.27 to 103.0	Yes	****	<0.0001	B-D
DSS vs. D	67.04	45.68 to 88.41	Yes	****	<0.0001	B-E
DSS-C100	22.92	1.558 to 44.28	Yes	*	0.0304	C-D
DSS-C100	8.329	-13.03 to 29.69	No	ns	0.7944	C-E
DSS-C200	-14.59	-35.95 to 6.771	No	ns	0.3044	D-E

Test detail	Mean 1	Mean 2	Mean Diff.	SE of diff.	n1	n2	q	DF
Control vs.	22.41	114.4	-91.97	7.43	8	8	17.5	35
Control vs.	22.41	55.66	-33.25	7.43	8	8	6.329	35
Control vs.	22.41	32.74	-10.33	7.43	8	8	1.967	35
Control vs.	22.41	47.33	-24.92	7.43	8	8	4.744	35
DSS vs. D	114.4	55.66	58.71	7.43	8	8	11.18	35
DSS vs. D	114.4	32.74	81.63	7.43	8	8	15.54	35
DSS vs. D	114.4	47.33	67.04	7.43	8	8	12.76	35
DSS-C100	55.66	32.74	22.92	7.43	8	8	4.363	35
DSS-C100	55.66	47.33	8.329	7.43	8	8	1.585	35
DSS-C200	32.74	47.33	-14.59	7.43	8	8	2.777	35

DSS-Cin-IL-1β-ELISA:

Control	DSS	DSS-Cin100	DSS-Cin200	DSS-SAZ
4.22	121.67	54.99	23.14	48.12
5.82	132.11	76.63	48.99	68.22
5.29	116.11	62.19	27.42	41.22
24.192	72.21	34.17	53.9	72.92
17.73	92.27	73.62	23.28	68.72
10.11	67.08	43.18	41.43	43.67
12.9	156.03	36.18	63.49	42.9
9.53	163.12	56.22	34.65	65.11

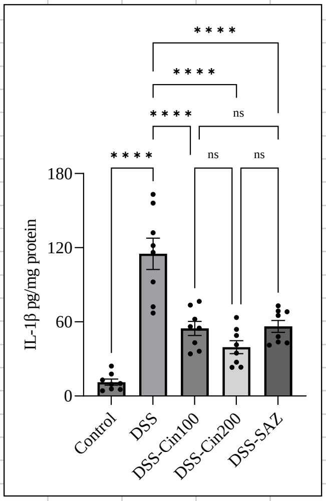

Table Anal	DSS-Cin-IL1B-ELISA
Data sets	A-E
ANOVA summary	
F	28.9
P value	<0.0001
P value su	****
Significant	Yes
R squared	0.7676

ANOVA ta	SS	DF	MS	F (DFn, DF	P value
Treatment	46126	4	11531	F (4, 35) =	P<0.0001
Residual (v	13967	35	399.1		
Total	60093	39			

Data summary	
Number of	5
Number of	40
Number of	1
Number of	10
Alpha	0.05

Tukey's mu	Mean Diff.	95.00% CI of	Below thresh	Summary	Adjusted P Value	
Control vs.	-103.9	-132.6 to -75.	Yes	****	<0.0001	A-B
Control vs.	-43.42	-72.14 to -14.	Yes	**	0.001	A-C
Control vs.	-28.31	-57.03 to 0.4(	No	ns	0.0549	A-D
Control vs.	-45.14	-73.85 to -16.	Yes	***	0.0006	A-E
DSS vs. D	60.43	31.71 to 89.1	Yes	****	<0.0001	B-C
DSS vs. D	75.54	46.82 to 104.	Yes	****	<0.0001	B-D
DSS vs. D	58.72	30.00 to 87.4	Yes	****	<0.0001	B-E
DSS-C100	15.11	-13.61 to 43.8	No	ns	0.5614	C-D
DSS-C100	-1.713	-30.43 to 27.(	No	ns	0.9998	C-E
DSS-C200	-16.82	-45.54 to 11.8	No	ns	0.4564	D-E

Test details	Mean 1	Mean 2	Mean Diff.	SE of diff.	n1	n2	q	DF
Control vs.	11.22	115.1	-103.9	9.988	8	8	14.7	35
Control vs.	11.22	54.65	-43.42	9.988	8	8	6.148	35
Control vs.	11.22	39.54	-28.31	9.988	8	8	4.009	35
Control vs.	11.22	56.36	-45.14	9.988	8	8	6.391	35
DSS vs. D	115.1	54.65	60.43	9.988	8	8	8.556	35
DSS vs. D	115.1	39.54	75.54	9.988	8	8	10.7	35
DSS vs. D	115.1	56.36	58.72	9.988	8	8	8.313	35
DSS-C100	54.65	39.54	15.11	9.988	8	8	2.139	35
DSS-C100	54.65	56.36	-1.713	9.988	8	8	0.2425	35
DSS-C200	39.54	56.36	-16.82	9.988	8	8	2.382	35

DSS-Cin-TNF-α-ELISA:

Control	DSS	DSS-C100	DSS-C200	DSS-SAZ
13.63225	89.04063	48.56348	46.68515	58.15767
16.01616	104.8936	60.49857	79.53854	51.27376
28.6264	63.928	45.49959	68.37832	48.43414
25.58958	212.7386	82.24087	36.32931	31.79357
11.98733	119.962	75.16086	12.0234	45.85912
15.76626	73.4782	37.01567	46.50057	32.49432
31.38969	98.05684	42.59628	32.99395	44.89788
41.11523	125.2567	59.13386	53.42176	82.49747

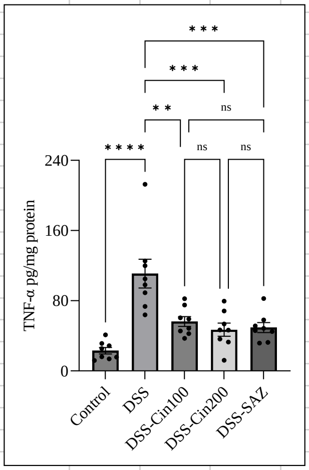

Table Anal	DSS-Cin-TNFa-ELISA
Data sets	A-E

ANOVA summary	
F	13.2
P value	<0.0001
P value su	****
Significant	Yes
R squared	0.6014

ANOVA ta	SS	DF	MS	F (DFn, DFd)	P value
Treatment	33758	4	8440	F (4, 35) = 13.20	P<0.0001
Residual (\	22378	35	639.4		
Total	56137	39			

Data summary	
Number of	5
Number of	40
Number of	1
Number of	10
Alpha	0.05

Tukey's mu	Mean Diff.	95.00% CI of diff.	Below thresho	Summary	Adjusted P	Value
Control vs.	-87.9	-124.3 to -51.55	Yes	****	<0.0001	A-B
Control vs.	-33.32	-69.67 to 3.026	No	ns	0.0854	A-C
Control vs.	-23.97	-60.32 to 12.38	No	ns	0.3384	A-D
Control vs.	-26.41	-62.76 to 9.939	No	ns	0.2475	A-E
DSS vs. D	54.58	18.23 to 90.93	Yes	**	0.0011	B-C
DSS vs. D	63.94	27.59 to 100.3	Yes	***	0.0001	B-D
DSS vs. D	61.49	25.14 to 97.84	Yes	***	0.0002	B-E
DSS-Cin10	9.355	-26.99 to 45.70	No	ns	0.9455	C-D
DSS-Cin10	6.913	-29.44 to 43.26	No	ns	0.9816	C-E
DSS-Cin20	-2.442	-38.79 to 33.91	No	ns	0.9997	D-E

Test detail	Mean 1	Mean 2	Mean Diff.	SE of diff.	n1	n2	q	DF
Control vs.	23.02	110.9	-87.9	12.64	8	8	9.833	35
Control vs.	23.02	56.34	-33.32	12.64	8	8	3.727	35
Control vs.	23.02	46.98	-23.97	12.64	8	8	2.681	35
Control vs.	23.02	49.43	-26.41	12.64	8	8	2.954	35
DSS vs. D	110.9	56.34	54.58	12.64	8	8	6.105	35
DSS vs. D	110.9	46.98	63.94	12.64	8	8	7.152	35
DSS vs. D	110.9	49.43	61.49	12.64	8	8	6.878	35
DSS-Cin10	56.34	46.98	9.355	12.64	8	8	1.046	35
DSS-Cin10	56.34	49.43	6.913	12.64	8	8	0.7732	35
DSS-Cin20	46.98	49.43	-2.442	12.64	8	8	0.2732	35

DSS-Cin-IL17A-ELISA:

Control	DSS	DSS-Cin100	DSS-Cin200	DSS-SAZ
115.2609	362.9922	140.8297	234.6595	285.0894
114.3605	412.6884	385.7506	97.6083	194.3788
138.0362	391.6265	236.4721	175.1243	214.019
100.7928	489.5732	237.1874	179.1059	190.3755
134.6219	396.937	201.8706	168.4805	177.6509
105.0378	248.0007	288.6219	156.2287	253.4768
119.003	448.7728	267.3442	143.889	210.227
141.0028	379.266	192.2883	118.3427	178.992

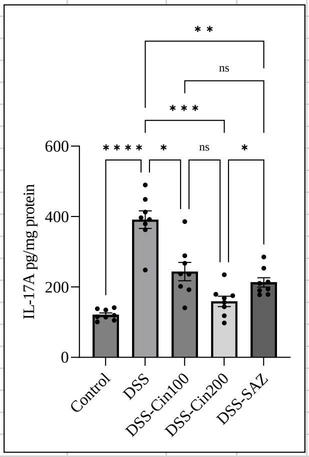

Table Anal	DSS-Cin-IL17-ELISA							
Repeated measures ANOVA summary								
Assume s	No							
F	27.19							
P value	<0.0001							
P value su	****							
Statisticall	Yes							
Geisser-Gr	0.5658							
R squared	0.7952							
ANOVA ta	SS	DF	MS	F (DFn, DFd)	P value			
Treatment	346183	4	86546	F (2.263, 15.84	P<0.0001			
Individual (	7488	7	1070	F (7, 28) = 0.33	P=0.9305			
Residual (r	89133	28	3183					
Total	442804	39						
Data summary								
Number of	5							
Number of	8							
Number of	0							
Number of	1							
Number of	10							
Alpha	0.05							
Tukey's m	Mean Diff.	95.00% CI of diff	Below threshold?	Summary	Adjusted P Value			
Control vs.	-270.2	-361.5 to -178.9	Yes	****	<0.0001	A-B		
Control vs.	-122.8	-224.0 to -21.51	Yes	*	0.02	A-C		
Control vs.	-38.17	-97.29 to 20.96	No	ns	0.2457	A-D		
Control vs.	-92.01	-150.5 to -33.52	Yes	**	0.0049	A-E		
DSS vs. D	147.4	19.56 to 275.3	Yes	*	0.0257	B-C		
DSS vs. D	232.1	125.3 to 338.8	Yes	***	0.0007	B-D		
DSS vs. D	178.2	55.50 to 300.9	Yes	**	0.0077	B-E		
DSS-Cin1(	84.62	-51.50 to 220.7	No	ns	0.2723	C-D		
DSS-Cin1(	30.77	-84.20 to 145.7	No	ns	0.8657	C-E		
DSS-Cin2(	-53.85	-96.54 to -11.15	Yes	*	0.0164	D-E		
Test detail	Mean 1	Mean 2	Mean Diff.	SE of diff.	n1	n2	q	DF
Control vs.	121	391.2	-270.2	25.51	8	8	14.98	7
Control vs.	121	243.8	-122.8	28.3	8	8	6.135	7
Control vs.	121	159.2	-38.17	16.52	8	8	3.266	7
Control vs.	121	213	-92.01	16.35	8	8	7.96	7
DSS vs. D	391.2	243.8	147.4	35.74	8	8	5.834	7
DSS vs. D	391.2	159.2	232.1	29.84	8	8	11	7
DSS vs. D	391.2	213	178.2	34.29	8	8	7.349	7
DSS-Cin1(	243.8	159.2	84.62	38.04	8	8	3.146	7
DSS-Cin1(	243.8	213	30.77	32.13	8	8	1.354	7
DSS-Cin2(	159.2	213	-53.85	11.93	8	8	6.382	7

DSS-Cin-IL6-mRNA:

Control	DSS	DSS-C100	DSS-C200	DSS-SAZ
1	5.122	4.3889	2.2278	3.1178
1	6.367	3.0776	3.1187	3.2289
1	7.377	3.5279	2.6533	1.1167
1	5.299	2.3889	2.1189	1.9983
1	4.889	3.1997	1.9822	3.7782
1	7.299	2.9008	1.45	2.22889

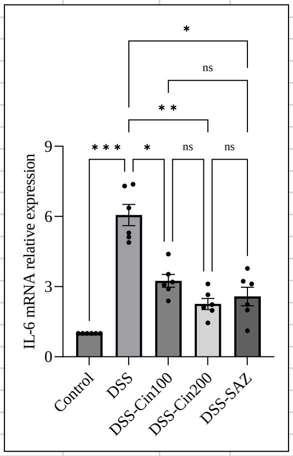

Table Anal	DSS-Cin-IL6-mRNA
Repeated measures ANOVA summary	
Assume s	No
F	32.55
P value	<0.0001
P value su	****
Statisticall	Yes
Geisser-Gr	0.4638
R squared	0.8669
Was the matching effective?	
F	0.5607
P value	0.7288
P value su	ns
Is there sig	No
R squared	0.01832

ANOVA ta	SS	DF	MS	F (DFn, DFd)	P value
Treatment	84.85	4	21.21	F (1.855, 9.27	P<0.0001
Individual (	1.827	5	0.3654	F (5, 20) = 0.5	P=0.7288
Residual (r	13.03	20	0.6516		
Total	99.71	29			

Data summary	
Number of	5
Number of	6
Number of	0
Number of	1
Number of	10
Alpha	0.05

Tukey's mu	Mean Diff.	95.00% CI of	Below threshold	Summary	Adjusted P	Value
Control vs.	-5.059	-6.882 to -3.2	Yes	***	0.0006	A-B
Control vs.	-2.247	-3.351 to -1.1	Yes	**	0.0024	A-C
Control vs.	-1.258	-2.198 to -0.3	Yes	*	0.0156	A-D
Control vs.	-1.578	-3.174 to 0.0	No	ns	0.0521	A-E
DSS vs. D	2.812	0.5647 to 5.0	Yes	*	0.0206	B-C
DSS vs. D	3.8	1.814 to 5.78	Yes	**	0.0032	B-D
DSS vs. D	3.481	0.3563 to 6.6	Yes	*	0.033	B-E
DSS-C100	0.9888	-0.3276 to 2.	No	ns	0.1336	C-D
DSS-C100	0.6692	-1.080 to 2.4	No	ns	0.5854	C-E
DSS-C200	-0.3196	-2.172 to 1.5	No	ns	0.9498	D-E

Test detail	Mean 1	Mean 2	Mean Diff.	SE of diff.	n1	n2	q	DF
Control vs.	1	6.059	-5.059	0.4544	6	6	15.74	5
Control vs.	1	3.247	-2.247	0.2751	6	6	11.55	5
Control vs.	1	2.258	-1.258	0.2343	6	6	7.598	5
Control vs.	1	2.578	-1.578	0.3979	6	6	5.609	5
DSS vs. D	6.059	3.247	2.812	0.5601	6	6	7.099	5
DSS vs. D	6.059	2.258	3.8	0.4953	6	6	10.85	5
DSS vs. D	6.059	2.578	3.481	0.7789	6	6	6.32	5
DSS-C100	3.247	2.258	0.9888	0.3281	6	6	4.261	5
DSS-C100	3.247	2.578	0.6692	0.4361	6	6	2.17	5
DSS-C200	2.258	2.578	-0.3196	0.4619	6	6	0.9788	5

DSS-Cin-IL-1β-mRNA:

Control	DSS	DSS-Cin100	DSS-Cin200	DSS-SAZ		
1	3.227	1.2278	1.6672	2.27738		
1	4.2289	2.3778	1.22776	3.19928		
1	4.9902	3.0007	1.8992	2.0087		
1	5.22897	2.00936	2.1126	1.7726		
1	3.71881	2.2278	2.0027	1.5889		
1	5.502	1.9982	1.72655	1.8829		

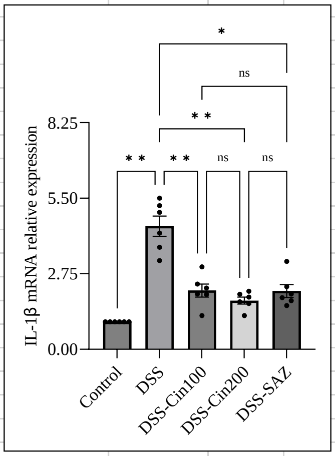

Table Anal	DSS-Cin-IL1b-mRNA					
Repeated measures ANOVA summary						
Assume s	No					
F	32.64					
P value	<0.0001					
P value su	****					
Statisticall	Yes					
Geisser-Gr	0.5758					
R squared	0.8672					
Was the matching effective?						
F	1.069					
P value	0.4068					
P value su	ns					
Is there sig	No					
R squared	0.03428					
ANOVA ta	SS	DF	MS	F (DFn, DFd)	P value	
Treatment	40.74	4	10.18	F (2.303, 11.5	P<0.0001	
Individual (	1.668	5	0.3335	F (5, 20) = 1.0	P=0.4068	
Residual (r	6.241	20	0.312			
Total	48.64	29				
Data summary						
Number of	5					
Number of	6					
Number of	0					
Number of	1					
Number of	10					
Alpha	0.05					
Tukey's mu	Mean Diff.	95.00% CI of diff	Below threshold?	Summary	Adjusted P	Value
Control vs.	-3.483	-4.962 to -2.004	Yes	**	0.0012	A-B
Control vs.	-1.14	-2.088 to -0.192	Yes	*	0.0242	A-C
Control vs.	-0.7727	-1.288 to -0.257	Yes	**	0.0096	A-D
Control vs.	-1.122	-2.066 to -0.177	Yes	*	0.0255	A-E
DSS vs. D	2.342	1.006 to 3.678	Yes	**	0.0048	B-C
DSS vs. D	2.71	1.275 to 4.145	Yes	**	0.0034	B-D
DSS vs. D	2.361	0.4253 to 4.297	Yes	*	0.0229	B-E
DSS-Cin10	0.3676	-0.6819 to 1.417	No	ns	0.6503	C-D
DSS-Cin10	0.01865	-1.299 to 1.336	No	ns	>0.9999	C-E
DSS-Cin20	-0.349	-1.786 to 1.088	No	ns	0.8567	D-E

DSS-Cin-TNFα-mRNA:

Control	DSS	DSS-Cin100	DSS-Cin200	DSS-SAZ
1	3.224	2.3776	1.8667	2.3773
1	4.1187	3.22891	1.7782	3.1189
1	5.2287	2.782	1.8455	3.0775
1	4.2289	2.0023	2.0889	2.0228
1	6.1997	3.9928	1.22718	1.6672
1	2.8897	1.9762	2.007	1.8892

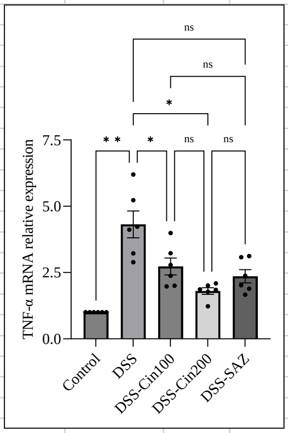

Table Anal	DSS-Cin-TNFa-mRNA
Repeated measures ANOVA summary	
Assume sp	No
F	18.52
P value	0.0015
P value su	**
Statisticall	Yes
Geisser-Gr	0.3935
R squared	0.7874
Was the matching effective?	
F	1.312
P value	0.2986
P value su	ns
Is there sig	No
R squared	0.0652

ANOVA ta	SS	DF	MS	F (DFn, DFd)	P value
Treatment	36.51	4	9.127	F (1.574, 7.871)	P=0.0015
Individual (	3.234	5	0.6468	F (5, 20) = 1.31	P=0.2986
Residual (r	9.857	20	0.4928		
Total	49.6	29			

Data summary	
Number of	5
Number of	6
Number of	0
Number of	1
Number of	10
Alpha	0.05

Tukey's mu	Mean Diff.	95.00% CI of d	Below threshold	Summary	Adjusted P	Value
Control vs.	-3.315	-5.341 to -1.28	Yes	**	0.0065	A-B
Control vs.	-1.727	-3.010 to -0.44	Yes	*	0.0152	A-C
Control vs.	-0.8022	-1.300 to -0.30	Yes	**	0.0069	A-D
Control vs.	-1.359	-2.370 to -0.34	Yes	*	0.0153	A-E
DSS vs. D	1.588	0.3154 to 2.86	Yes	*	0.0209	B-C
DSS vs. D	2.513	0.08705 to 4.9	Yes	*	0.0438	B-D
DSS vs. D	1.956	-0.3363 to 4.2	No	ns	0.0882	B-E
DSS-Cin10	0.9244	-0.8353 to 2.6	No	ns	0.3413	C-D
DSS-Cin10	0.3678	-1.221 to 1.95	No	ns	0.8747	C-E
DSS-Cin20	-0.5566	-1.574 to 0.46	No	ns	0.3122	D-E

Test detail	Mean 1	Mean 2	Mean Diff.	SE of diff.	n1	n2	q	DF
Control vs.	1	4.315	-3.315	0.5051	6	6	9.281	5
Control vs.	1	2.727	-1.727	0.3198	6	6	7.635	5
Control vs.	1	1.802	-0.8022	0.124	6	6	9.148	5
Control vs.	1	2.359	-1.359	0.2521	6	6	7.623	5
DSS vs. D	4.315	2.727	1.588	0.3173	6	6	7.079	5
DSS vs. D	4.315	1.802	2.513	0.6047	6	6	5.877	5
DSS vs. D	4.315	2.359	1.956	0.5715	6	6	4.841	5
DSS-Cin10	2.727	1.802	0.9244	0.4387	6	6	2.98	5
DSS-Cin10	2.727	2.359	0.3678	0.396	6	6	1.314	5
DSS-Cin20	1.802	2.359	-0.5566	0.2536	6	6	3.104	5

DSS-Cin-IL17A-mRNA:

Control	DSS	DSS-Cin100	DSS-Cin200	DSS-SAZ
1	4.226	3.2298	1.88276	2.3778
1	5.3387	2.3778	1.66773	3.27783
1	6.3889	2.8836	2.00938	3.99287
1	4.3387	3.9987	2.2276	1.9997
1	3.9982	2.2356	1.47728	1.366
1	4.9983	1.9927	1.3889	1.667

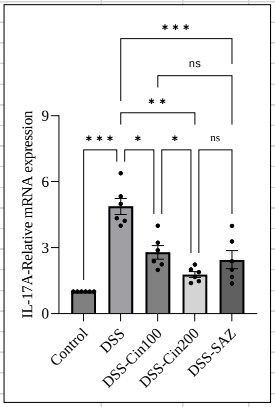

Table Anal	DSS-Cin-IL17-mRNA
Repeated measures ANOVA summary	
Assume s|	No
F	33.61
P value	0.0002
P value su	***
Statisticall	Yes
Geisser-G|	0.4023
R squared	0.8705
Was the matching effective?	
F	2.514
P value	0.0638
P value su	ns
Is there si	No
R squared	0.07528

ANOVA ta	SS	DF	MS	F (DFn, DFd)	P value
Treatment	51	4	12.75	F (1.609, 8.0	P=0.0002
Individual (	4.77	5	0.9539	F (5, 20) = 2.	P=0.0638
Residual (r	7.588	20	0.3794		
Total	63.36	29			

Data summary	
Number of	5
Number of	6
Number of	0
Number of	1
Number of	10
Alpha	0.05

Tukey's m	Mean Diff.	95.00% CI of dif	Below threshold?	Summary	Adjusted P	Value
Control vs.	-3.881	-5.346 to -2.417	Yes	***	0.0007	A-B
Control vs.	-1.786	-3.007 to -0.566	Yes	*	0.0106	A-C
Control vs.	-0.7756	-1.304 to -0.247	Yes	*	0.0105	A-D
Control vs.	-1.447	-3.095 to 0.2009	No	ns	0.08	A-E
DSS vs. D	2.095	0.02800 to 4.16	Yes	*	0.0475	B-C
DSS vs. D	3.106	1.621 to 4.590	Yes	**	0.0021	B-D
DSS vs. D	2.435	1.588 to 3.282	Yes	***	0.0005	B-E
DSS-Cin10	1.011	0.2676 to 1.754	Yes	*	0.0146	C-D
DSS-Cin10	0.3395	-1.589 to 2.268	No	ns	0.9464	C-E
DSS-Cin20	-0.6713	-2.174 to 0.8315	No	ns	0.4649	D-E

Test detail	Mean 1	Mean 2	Mean Diff.	SE of diff.	n1	n2	q	DF
Control vs.	1	4.881	-3.881	0.3651	6	6	15.03	5
Control vs.	1	2.786	-1.786	0.3042	6	6	8.305	5
Control vs.	1	1.776	-0.7756	0.1317	6	6	8.327	5
Control vs.	1	2.447	-1.447	0.4108	6	6	4.981	5
DSS vs. D	4.881	2.786	2.095	0.5153	6	6	5.75	5
DSS vs. D	4.881	1.776	3.106	0.3701	6	6	11.87	5
DSS vs. D	4.881	2.447	2.435	0.2111	6	6	16.31	5
DSS-Cin10	2.786	1.776	1.011	0.1853	6	6	7.716	5
DSS-Cin10	2.786	2.447	0.3395	0.4807	6	6	0.9987	5
DSS-Cin20	1.776	2.447	-0.6713	0.3746	6	6	2.534	5

DSS-Cin-COX2 protein and mRNA:

Control	DSS	DSS-Cin100	DSS-Cin200
0.547493	0.8365	0.621161	0.549954
0.414008	1.0869	0.725195	0.661311
0.327196	0.899796	0.52233	0.42436

Protein

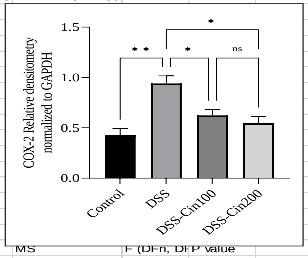

Table Anal	DSS-Cin-COX2-Protein
Data sets	A-D
ANOVA summary	
F	10.75
P value	0.0035
P value su	**
Significant	Yes
R squared	0.8013

ANOVA ta	SS	DF	MS	F (DFn, DF	P value
Treatment	0.4323	3	0.1441	F (3, 8) = 1	P=0.0035
Residual (v	0.1072	8	0.0134		
Total	0.5395	11			

Data summary	
Number of	4
Number of	12
Number of	1
Number of	6
Alpha	0.05

Tukey's m	Mean Diff.	95.00% CI of d	Below threshold?	Summary	Adjusted P	Value
Control vs.	-0.5115	-0.8142 to -0.2	Yes	**	0.0028	A-B
Control vs.	-0.1933	-0.4960 to 0.10	No	ns	0.249	A-C
Control vs.	-0.1156	-0.4183 to 0.18	No	ns	0.6305	A-D
DSS vs. D	0.3182	0.01547 to 0.62	Yes	*	0.0397	B-C
DSS vs. D	0.3959	0.09315 to 0.69	Yes	*	0.013	B-D
DSS-Cin10	0.07769	-0.2250 to 0.38	No	ns	0.8427	C-D

Test detail	Mean 1	Mean 2	Mean Diff.	SE of diff.	n1	n2	q	DF
Control vs.	0.4296	0.9411	-0.5115	0.09453	3	3	7.653	8
Control vs.	0.4296	0.6229	-0.1933	0.09453	3	3	2.892	8
Control vs.	0.4296	0.5452	-0.1156	0.09453	3	3	1.73	8
DSS vs. D	0.9411	0.6229	0.3182	0.09453	3	3	4.76	8
DSS vs. D	0.9411	0.5452	0.3959	0.09453	3	3	5.922	8
DSS-Cin10	0.6229	0.5452	0.07769	0.09453	3	3	1.162	8

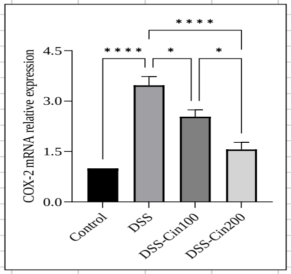

	Control	DSS	DSS-Cin100	DSS-Cin200
**mRNA**	1	4	3	2.1
	1	3.66	2.16	2.13
	1	4.23	3.11	1.59
	1	3.35	2.82	1.56
	1	2.45	2.19	0.82
	1	3.13	1.93	1.17

Table Anal	DSS-Cin COX2-RNA
Data sets	A-D
ANOVA summary	
F	30.71
P value	<0.0001
P value su	****
Significant	Yes
R squared	0.8216

ANOVA ta	SS	DF	MS	F (DFn, DF	P value
Treatment	21.35	3	7.118	F (3, 20) =	P<0.0001
Residual (v	4.636	20	0.2318		
Total	25.99	23			

Data summary	
Number of	4
Number of	24
Number of	1
Number of	6
Alpha	0.05

Tukey's m	Mean Diff.	95.00% CI of d	Below threshold?	Summary	Adjusted P	Value
Control vs.	-2.47	-3.248 to -1.69	Yes	****	<0.0001	A-B
Control vs.	-1.535	-2.313 to -0.75	Yes	***	0.0001	A-C
Control vs.	-0.5617	-1.340 to 0.216	No	ns	0.2138	A-D
DSS vs. D	0.935	0.1570 to 1.71	Yes	*	0.015	B-C
DSS vs. D	1.908	1.130 to 2.686	Yes	****	<0.0001	B-D
DSS-Cin10	0.9733	0.1953 to 1.75	Yes	*	0.0111	C-D

Test detail	Mean 1	Mean 2	Mean Diff.	SE of diff.	n1	n2	q	DF
Control vs.	1	3.47	-2.47	0.278	6	6	12.57	20
Control vs.	1	2.535	-1.535	0.278	6	6	7.81	20
Control vs.	1	1.562	-0.5617	0.278	6	6	2.858	20
DSS vs. D	3.47	2.535	0.935	0.278	6	6	4.757	20
DSS vs. D	3.47	1.562	1.908	0.278	6	6	9.709	20
DSS-Cin10	2.535	1.562	0.9733	0.278	6	6	4.952	20

DSS-Cin-Keap1, Nrf2 protein:

Control	DSS	DSS-Cin100	DSS-Cin200
0.658177	1.16595	0.918419	0.972059
0.489079	0.994904	0.609535	0.873918
0.481106	0.913083	0.966696	0.745264

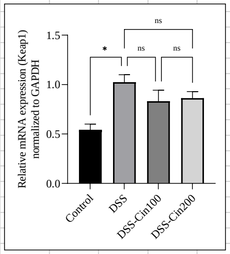

Keap-1 Western

Table Anal	DSS-Cin-Keap1								
Data sets	A-D								
ANOVA summary									
F	6.259								
P value	0.0171								
P value su	*								
Significant	Yes								
R squared	0.7012								
ANOVA ta	SS	DF	MS	F (DFn, DF	P value				
Treatment	0.3621	3	0.1207	F (3, 8) = (	P=0.0171				
Residual (\	0.1543	8	0.01928						
Total	0.5164	11							
Data summary									
Number of	4								
Number of	12								
Number of	1								
Number of	6								
Alpha	0.05								
Tukey's mı	Mean Diff.	95.00% CI of diff.	Below threshold?	Summary	Adjusted P Value				
Control vs.	-0.4819	-0.8450 to -0.1188	Yes	*	0.012	A-B			
Control vs.	-0.2888	-0.6519 to 0.07434	No	ns	0.1259	A-C			
Control vs.	-0.321	-0.6841 to 0.04214	No	ns	0.0845	A-D			
DSS vs. D	0.1931	-0.1700 to 0.5562	No	ns	0.3817	B-C			
DSS vs. D	0.1609	-0.2022 to 0.5240	No	ns	0.5227	B-D			
DSS-Cin1(	-0.0322	-0.3953 to 0.3309	No	ns	0.9914	C-D			
Test detail	Mean 1	Mean 2	Mean Diff.	SE of diff.	n1	n2	q	DF	
Control vs.	0.5428	1.025	-0.4819	0.1134	3	3	6.01	8	
Control vs.	0.5428	0.8316	-0.2888	0.1134	3	3	3.602	8	
Control vs.	0.5428	0.8637	-0.321	0.1134	3	3	4.003	8	
DSS vs. D	1.025	0.8316	0.1931	0.1134	3	3	2.408	8	
DSS vs. D	1.025	0.8637	0.1609	0.1134	3	3	2.007	8	
DSS-Cin1(	0.8316	0.8637	-0.0322	0.1134	3	3	0.4016	8	

Cytosolic Nrf2

Control	DSS	DSS-Cin100	DSS-Cin200
0.598672	0.554152	0.798468	0.852123
0.652107	0.596741	0.799028	0.89308
0.426105	0.254502	0.45372	0.651349
0.486865	0.207344	0.304157	0.748009

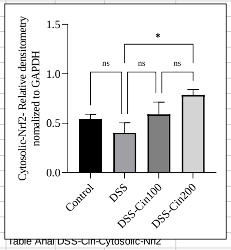

Table Anal	DSS-Cin-Cytosolic-Nrf2								
Data sets	A-D								
ANOVA summary									
F	3.212								
P value	0.0617								
P value su	ns								
Significant	No								
R squared	0.4454								
ANOVA ta	SS	DF	MS	F (DFn, DF	P value				
Treatment	0.3014	3	0.1005	F (3, 12) =	P=0.0617				
Residual (\	0.3754	12	0.03128						
Total	0.6768	15							
Data summary									
Number of	4								
Number of	16								
Number of	1								
Number of	6								
Alpha	0.05								
Tukey's mı	Mean Diff.	95.00% CI of di	Below threshold?	Summary	Adjusted P Value				
Control vs.	0.1378	-0.2335 to 0.50	No	ns	0.6954				
Control vs.	-0.04791	-0.4192 to 0.32	No	ns	0.98				
Control vs.	-0.2452	-0.6165 to 0.12	No	ns	0.2553				
DSS vs. D	-0.1857	-0.5569 to 0.18	No	ns	0.4756				
DSS vs. D	-0.383	-0.7542 to -0.01	Yes	*	0.0425				
DSS-Cin1(	-0.1973	-0.5686 to 0.17	No	ns	0.426	C-D			
Test detail	Mean 1	Mean 2	Mean Diff.	SE of diff.	n1	n2	q	DF	
Control vs.	0.5409	0.4032	0.1378	0.1251	4	4	1.558	12	
Control vs.	0.5409	0.5888	-0.04791	0.1251	4	4	0.5417	12	
Control vs.	0.5409	0.7861	-0.2452	0.1251	4	4	2.773	12	
DSS vs. D	0.4032	0.5888	-0.1857	0.1251	4	4	2.099	12	
DSS vs. D	0.4032	0.7861	-0.383	0.1251	4	4	4.331	12	
DSS-Cin1(	0.5888	0.7861	-0.1973	0.1251	4	4	2.231	12	

Nuclear Nrf2

Control	DSS	DSS-Cin100	DSS-Cin200
0.44442	0.269811	0.755048	1.858767
0.436189	0.568749	0.867555	1.66965
0.357631	0.563747	0.784555	1.834917
0.473034	0.469346	0.767393	1.166955

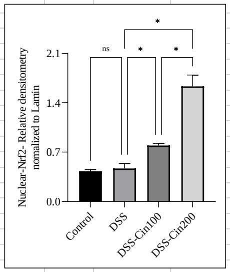

Table Anal	DSS-Cin-Nuclear-Nrf2								
Repeated measures ANOVA summary									
Assume s	No								
F	36.29								
P value	0.0034								
P value su	**								
Statisticall	Yes								
Geisser-Gı	0.4398								
R squared	0.9236								
Was the matching effective?									
F	0.7123								
P value	0.5688								
P value su	ns								
Is there sig	No								
R squared	0.01781								
ANOVA ta	SS	DF	MS	F (DFn, DF	P value				
Treatment	3.753	3	1.251	F (1.319, 3	P=0.0034				
Individual (	0.07367	3	0.02456	F (3, 9) = (	P=0.5688				
Residual (r	0.3103	9	0.03447						
Total	4.137	15							
Data summary									
Number of	4								
Number of	4								
Number of	0								
Number of	1								
Number of	6								
Alpha	0.05								
Tukey's mı	Mean Diff.	95.00% CI	Below thre	Summary	Adjusted P Value				
Control vs.	-0.04009	-0.4441 to	No	ns	0.9589	A-B			
Control vs.	-0.3658	-0.5430 to	Yes	**	0.0065	A-C			
Control vs.	-1.205	-2.063 to -(	Yes	*	0.0198	A-D			
DSS vs. D	-0.3257	-0.5971 to	Yes	*	0.0305	B-C			
DSS vs. D	-1.165	-2.061 to -(	Yes	*	0.0245	B-D			
DSS-Cin1(	-0.8389	-1.614 to -(	Yes	*	0.0404	C-D			
Test detail	Mean 1	Mean 2	Mean Diff.	SE of diff.	n1	n2	q	DF	
Control vs.	0.4278	0.4679	-0.04009	0.08373	4	4	0.6772	3	
Control vs.	0.4278	0.7936	-0.3658	0.03672	4	4	14.09	3	
Control vs.	0.4278	1.633	-1.205	0.1779	4	4	9.575	3	
DSS vs. D	0.4679	0.7936	-0.3257	0.05623	4	4	8.192	3	
DSS vs. D	0.4679	1.633	-1.165	0.1856	4	4	8.872	3	
DSS-Cin1(	0.7936	1.633	-0.8389	0.1605	4	4	7.391	3	

DSS-Cin-NQO1, HO1 protein & mRNA:

NQO1-Western

Control	DSS	DSS-Cin100	DSS-Cin200
0.876514	0.588963	0.551396	0.90663
0.857208	0.519801	0.504068	0.688507
0.604483	0.356882	0.450949	0.674108
0.981146	0.330014	0.911198	0.866003

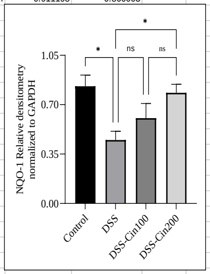

Table Anal	DSS-Cin-NQO1-Western
Data sets	A-D
ANOVA summary	
F	4.931
P value	0.0186
P value su	*
Significant	Yes
R squared	0.5521

ANOVA ta	SS	DF	MS	F (DFn, DF	P value
Treatment	0.3666	3	0.1222	F (3, 12) =	P=0.0186
Residual (v	0.2974	12	0.02478		
Total	0.6639	15			

Data summary	
Number of	4
Number of	16
Number of	1
Number of	6
Alpha	0.05

Tukey's mu	Mean Diff.	95.00% CI of diff	Below threshold?	Summary	Adjusted P Value	
Control vs.	0.3809	0.05045 to 0.711	Yes	*	0.0227	A-B
Control vs.	0.2254	-0.1050 to 0.555	No	ns	0.2324	A-C
Control vs.	0.04603	-0.2844 to 0.376	No	ns	0.9751	A-D
DSS vs. D	-0.1555	-0.4860 to 0.175	No	ns	0.5244	B-C
DSS vs. D	-0.3349	-0.6654 to -0.004	Yes	*	0.0467	B-D
DSS-Cin10	-0.1794	-0.5099 to 0.151	No	ns	0.4085	C-D

Test detail	Mean 1	Mean 2	Mean Diff.	SE of diff.	n1	n2	q	DF
Control vs.	0.8298	0.4489	0.3809	0.1113	4	4	4.84	12
Control vs.	0.8298	0.6044	0.2254	0.1113	4	4	2.864	12
Control vs.	0.8298	0.7838	0.04603	0.1113	4	4	0.5848	12
DSS vs. D	0.4489	0.6044	-0.1555	0.1113	4	4	1.975	12
DSS vs. D	0.4489	0.7838	-0.3349	0.1113	4	4	4.255	12
DSS-Cin10	0.6044	0.7838	-0.1794	0.1113	4	4	2.279	12

	Control	DSS	DSS-Cin10	DSS-Cin200
NQO1-mR	1	0.72	1.11	1.5002
	1	0.66	0.9166	2.124
	1	0.68	0.9	1.887
	1	0.55	0.5874	2.11
	1	0.61	1.1	1.89
	1	0.45	0.792	1.8833

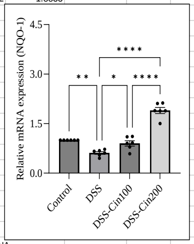

Table Anal	DSS-Cin-NQO1-mRNA
Data sets	A-D
ANOVA summary	
F	74.56
P value	<0.0001
P value su	****
Significant	Yes
R squared	0.9179

ANOVA ta	SS	DF	MS	F (DFn, DFd)	P value
Treatment	5.559	3	1.853	F (3, 20) = 74.5	P<0.0001
Residual (v	0.4971	20	0.02485		
Total	6.057	23			

Data summary	
Number of	4
Number of	24
Number of	1
Number of	6
Alpha	0.05

Tukey's mu	Mean Diff.	95.00% CI	Below thresho	Summary	Adjusted P Value	
Control vs.	0.3883	0.1336 to (	Yes	**	0.002	A-B
Control vs.	0.099	-0.1558 to	No	ns	0.7009	A-C
Control vs.	-0.8991	-1.154 to -(	Yes	****	<0.0001	A-D
DSS vs. D	-0.2893	-0.5441 to	Yes	*	0.0225	B-C
DSS vs. D	-1.287	-1.542 to -	Yes	****	<0.0001	B-D
DSS-Cin10	-0.9981	-1.253 to -(	Yes	****	<0.0001	C-D

Test detail	Mean 1	Mean 2	Mean Diff.	SE of diff.	n1	n2	q	DF
Control vs.	1	0.6117	0.3883	0.09102	6	6	6.034	20
Control vs.	1	0.901	0.099	0.09102	6	6	1.538	20
Control vs.	1	1.899	-0.8991	0.09102	6	6	13.97	20
DSS vs. D	0.6117	0.901	-0.2893	0.09102	6	6	4.495	20
DSS vs. D	0.6117	1.899	-1.287	0.09102	6	6	20	20
DSS-Cin10	0.901	1.899	-0.9981	0.09102	6	6	15.51	20

	Control	DSS	DSS-Cin100	DSS-Cin200
**HO1-Western**	1.011109	0.308428	0.582038	0.932501
	0.85943	0.416965	0.474177	0.705512
	0.867602	0.520347	0.631576	0.809086

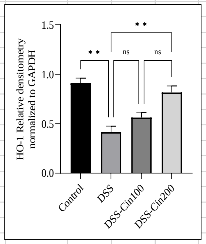

Table Anal	DSS-Cin-HO1-Western
Data sets	A-D
ANOVA summary	
F	16.51
P value	0.0009
P value su	***
Significant	Yes
R squared	0.8609

ANOVA ta	SS	DF	MS	F (DFn, DF	P value
Treatment	0.4692	3	0.1564	F (3, 8) = 1	P=0.0009
Residual (v	0.0758	8	0.009475		
Total	0.545	11			

Data summary	
Number of	4
Number of	12
Number of	1
Number of	6
Alpha	0.05

Tukey's mu	Mean Diff.	95.00% CI of (	Below threshol	Summary	Adjusted P Value	
Control vs.	0.4975	0.2430 to 0.75	Yes	**	0.0011	A-B
Control vs.	0.3501	0.09561 to 0.6	Yes	**	0.0098	A-C
Control vs.	0.09701	-0.1575 to 0.3	No	ns	0.632	A-D
DSS vs. D	-0.1474	-0.4019 to 0.1	No	ns	0.3178	B-C
DSS vs. D	-0.4005	-0.6550 to -0.1	Yes	**	0.0044	B-D
DSS-Cin10	-0.2531	-0.5076 to 0.0	No	ns	0.0513	C-D

Test detail	Mean 1	Mean 2	Mean Diff.	SE of diff.	n1	n2	q	DF
Control vs.	0.9127	0.4152	0.4975	0.07948	3	3	8.852	8
Control vs.	0.9127	0.5626	0.3501	0.07948	3	3	6.23	8
Control vs.	0.9127	0.8157	0.09701	0.07948	3	3	1.726	8
DSS vs. D	0.4152	0.5626	-0.1474	0.07948	3	3	2.622	8
DSS vs. D	0.4152	0.8157	-0.4005	0.07948	3	3	7.126	8
DSS-Cin10	0.5626	0.8157	-0.2531	0.07948	3	3	4.504	8

	Control	DSS	DSS-Cin100	DSS-Cin200
	1	0.84	1.882	2.9002
**HO1-mRN**	1	0.886	1.566	3.124
	1	0.5667	1.277	2.288
	1	0.75	1.2209	3.7566
	1	0.9553	2.366	3.15
	1	0.66	0.8276	2.7833

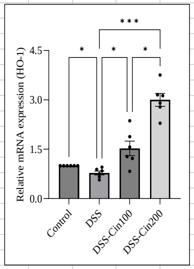

Table Anal	DSS-Cin-HO1-mRNA
Repeated measures ANOVA summary	
Assume s	No
F	52.45
P value	<0.0001
P value su	****
Statisticall	Yes
Geisser-G	0.6054
R squared	0.913

ANOVA ta	SS	DF	MS	F (DFn, DF	P value
Treatment	18.02	3	6.006	F (1.816, 9	P<0.0001
Individual (	1.04	5	0.2079	F (5, 15) =	P=0.1702
Residual (r	1.718	15	0.1145		
Total	20.77	23			

Data summary	
Number of	4
Number of	6
Number of	0
Number of	1
Number of	6
Alpha	0.05

Tukey's mu	Mean Diff.	95.00% CI of diff.	Below thre	Summary	Adjusted P Value	
Control vs.	0.2237	0.004040 to 0.4433	Yes	*	0.0468	A-B
Control vs.	-0.5233	-1.342 to 0.2954	No	ns	0.2036	A-C
Control vs.	-2	-2.730 to -1.270	Yes	***	0.0006	A-D
DSS vs. D	-0.7469	-1.402 to -0.09192	Yes	*	0.0305	B-C
DSS vs. D	-2.224	-2.864 to -1.584	Yes	***	0.0002	B-D
DSS-Cin10	-1.477	-2.491 to -0.4633	Yes	*	0.0112	C-D

Test detail	Mean 1	Mean 2	Mean Diff.	SE of diff.	n1	n2	q	DF
Control vs.	1	0.7763	0.2237	0.05952	6	6	5.314	5
Control vs.	1	1.523	-0.5233	0.2219	6	6	3.335	5
Control vs.	1	3	-2	0.1978	6	6	14.3	5
DSS vs. D	0.7763	1.523	-0.7469	0.1775	6	6	5.951	5
DSS vs. D	0.7763	3	-2.224	0.1735	6	6	18.13	5
DSS-Cin10	1.523	3	-1.477	0.2747	6	6	7.603	5

DSS-Cin-PPARγ, PPARα, PPARβ/δ protein:

DSS	DSS-Cin100	DSS-Cin200
0.522121	0.400706	1.353748
0.814931	0.780394	1.27904
0.528598	0.644656	0.982201
0.412681	0.813928	1.30953

estern

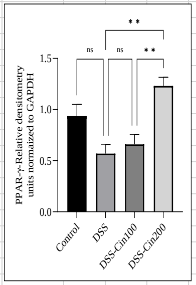

DSS-Cin-PPARg	
A-D	
ummary	
	9.73
	0.0016
**	
Yes	
	0.7087

SS	DF	MS	F (DFn, DF	P value
1.069	3	0.3564	F (3, 12) =	P=0.0016
0.4396	12	0.03663		
1.509	15			

nary
4
16
1
6
0.05

Mean Diff.	95.00% CI of diff	Below threshold	Summary	Adjusted P Value	
0.3659	-0.03590 to 0.76	No	ns	0.0787	A-B
0.2756	-0.1262 to 0.677	No	ns	0.2287	A-C
-0.2956	-0.6974 to 0.106	No	ns	0.1827	A-D
-0.09034	-0.4921 to 0.311	No	ns	0.9073	B-C
-0.6615	-1.063 to -0.2597	Yes	**	0.0018	B-D
-0.5712	-0.9730 to -0.169	Yes	**	0.0056	C-D

Mean 1	Mean 2	Mean Diff.	SE of diff.	n1	n2	q	DF
0.9355	0.5696	0.3659	0.1353	4	4	3.824	12
0.9355	0.6599	0.2756	0.1353	4	4	2.88	12
0.9355	1.231	-0.2956	0.1353	4	4	3.089	12
0.5696	0.6599	-0.09034	0.1353	4	4	0.944	12
0.5696	1.231	-0.6615	0.1353	4	4	6.913	12
0.6599	1.231	-0.5712	0.1353	4	4	5.969	12

PPAR-alpha

Control	DSS	DSS-Cin100	DSS-Cin200
0.741209	0.54746	0.588347	0.704257
0.567483	0.542467	0.579574	0.58129
0.640672	0.49468	0.530837	0.708043
0.442898	0.605322	0.4063	0.466134
0.578037	0.695512	0.592535	0.679783

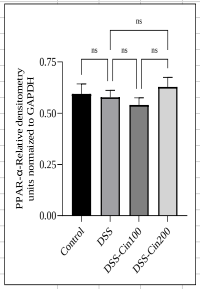

Table Anal	DSS-Cin-PPARalpha
Data sets	A-D
ANOVA summary	
F	0.7763
P value	0.5242
P value su	ns
Significant	No
R squared	0.1271

ANOVA ta	SS	DF	MS	F (DFn, DF	P value
Treatment	0.02027	3	0.006755	F (3, 16) =	P=0.5242
Residual (\	0.1392	16	0.008702		
Total	0.1595	19			

Data summary	
Number of	4
Number of	20
Number of	1
Number of	6
Alpha	0.05

Tukey's mu	Mean Diff.	95.00% CI of di	Below threshold?	Summary	Adjusted P Value	
Control vs.	0.01697	-0.1518 to 0.18	No	ns	0.9914	A-B
Control vs.	0.05454	-0.1143 to 0.22	No	ns	0.7923	A-C
Control vs.	-0.03384	-0.2026 to 0.13	No	ns	0.9385	A-D
DSS vs. D	0.03757	-0.1312 to 0.20	No	ns	0.9186	B-C
DSS vs. D	-0.05081	-0.2196 to 0.11	No	ns	0.8244	B-D
DSS-Cin10	-0.08838	-0.2572 to 0.08	No	ns	0.4614	C-D

Test detail	Mean 1	Mean 2	Mean Diff.	SE of diff.	n1	n2	q	DF
Control vs.	0.5941	0.5771	0.01697	0.059	5	5	0.4068	16
Control vs.	0.5941	0.5395	0.05454	0.059	5	5	1.307	16
Control vs.	0.5941	0.6279	-0.03384	0.059	5	5	0.8112	16
DSS vs. D	0.5771	0.5395	0.03757	0.059	5	5	0.9005	16
DSS vs. D	0.5771	0.6279	-0.05081	0.059	5	5	1.218	16
DSS-Cin10	0.5395	0.6279	-0.08838	0.059	5	5	2.119	16

PPAR-delta

Control	DSS	DSS-Cin100	DSS-Cin200
0.534566	0.58289	0.568228	0.673288
0.500064	0.326451	0.344069	0.361617
0.395876	0.537639	0.554202	0.591881
0.394177	0.496777	0.223552	0.363484
0.561204	0.583546	0.350512	0.400839

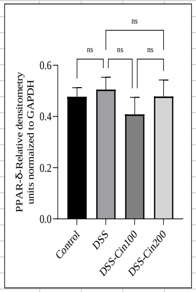

Table Anal	DSS-Cin-PPARdelta
Data sets	A-D
ANOVA summary	
F	0.5708
P value	0.6423
P value su	ns
Significant	No
R squared	0.09668

ANOVA ta	SS	DF	MS	F (DFn, DF	P value
Treatment	0.02588	3	0.008627	F (3, 16) =	P=0.6423
Residual (\	0.2418	16	0.01511		
Total	0.2677	19			

Data summary	
Number of	4
Number of	20
Number of	1
Number of	6
Alpha	0.05

Tukey's mu	Mean Diff.	95.00% CI of	Below threshold	Summary	Adjusted P Value	
Control vs.	-0.02828	-0.2507 to 0.1	No	ns	0.9829	A-B
Control vs.	0.06906	-0.1534 to 0.2	No	ns	0.8109	A-C
Control vs.	-0.00104	-0.2235 to 0.2	No	ns	>0.9999	A-D
DSS vs. D	0.09735	-0.1251 to 0.3	No	ns	0.6045	B-C
DSS vs. D	0.02724	-0.1952 to 0.2	No	ns	0.9847	B-D
DSS-Cin10	-0.07011	-0.2926 to 0.1	No	ns	0.8041	C-D

Test detail	Mean 1	Mean 2	Mean Diff.	SE of diff.	n1	n2	q	DF
Control vs.	0.4772	0.5055	-0.02828	0.07775	5	5	0.5144	16
Control vs.	0.4772	0.4081	0.06906	0.07775	5	5	1.256	16
Control vs.	0.4772	0.4782	-0.001044	0.07775	5	5	0.019	16
DSS vs. D	0.5055	0.4081	0.09735	0.07775	5	5	1.771	16
DSS vs. D	0.5055	0.4782	0.02724	0.07775	5	5	0.4955	16
DSS-Cin10	0.4081	0.4782	-0.07011	0.07775	5	5	1.275	16

pNFkB-p6

Control	DSS	DSS-Cin100	DSS-Cin200
0.760336	2.023419	0.908163	0.771938
0.635876	1.860608	1.086084	0.535503
0.690271	1.730153	1.293019	0.680133
0.525403	1.921856	1.506069	0.621328
0.575939	1.921224	1.321885	0.729982

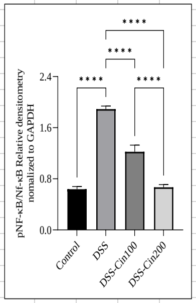

Table Anal	DSS-Cin-NF-kB-Western
Data sets	A-D
ANOVA summary	
F	84.82
P value	<0.0001
P value su	****
Significant	Yes
R squared	0.9408

ANOVA ta	SS	DF	MS	F (DFn, DF	P value
Treatment	5.211	3	1.737	F (3, 16) =	P<0.0001
Residual (\	0.3276	16	0.02048		
Total	5.538	19			

Data summary	
Number of	4
Number of	20
Number of	1
Number of	6
Alpha	0.05

Tukey's mu	Mean Diff.	95.00% CI of d	Below threshold	Summary	Adjusted P Value	
Control vs.	-1.254	-1.513 to -0.99	Yes	****	<0.0001	A-B
Control vs.	-0.5855	-0.8444 to -0.3	Yes	****	<0.0001	A-C
Control vs.	-0.03021	-0.2891 to 0.22	No	ns	0.9867	A-D
DSS vs. D	0.6684	0.4095 to 0.92	Yes	****	<0.0001	B-C
DSS vs. D	1.224	0.9647 to 1.48	Yes	****	<0.0001	B-D
DSS-Cin10	0.5553	0.2963 to 0.81	Yes	****	<0.0001	C-D

Test detail	Mean 1	Mean 2	Mean Diff.	SE of diff.	n1	n2	q	DF
Control vs.	0.6376	1.891	-1.254	0.0905	5	5	19.59	16
Control vs.	0.6376	1.223	-0.5855	0.0905	5	5	9.149	16
Control vs.	0.6376	0.6678	-0.03021	0.0905	5	5	0.4721	16
DSS vs. D	1.891	1.223	0.6684	0.0905	5	5	10.44	16
DSS vs. D	1.891	0.6678	1.224	0.0905	5	5	19.12	16
DSS-Cin10	1.223	0.6678	0.5553	0.0905	5	5	8.677	16

HT-29-Cell viability, IL8 & CXCL-1 mRNA:

3.12	95.89305	97.63231	96.76268	100.691	96.35874	98.52487
6.25	90.58902	99.91226	95.25064	99.70557	97.03281	98.36919
12.5	95.03353	93.93311	94.48332	92.00751	92.90316	92.45533
25	97.24092	95.2905	96.26571	88.23722	88.21058	88.2239
50	94.37744	94.02709	94.20227	94.83103	90.62144	92.72624
100	93.59823	94.49998	94.0491	89.05677	92.19644	90.6266
200	81.63992	86.56029	84.10011	80.51276	83.00788	81.76032
	96.80729	96.53011	96.6687	95.26889	95.71131	95.4901

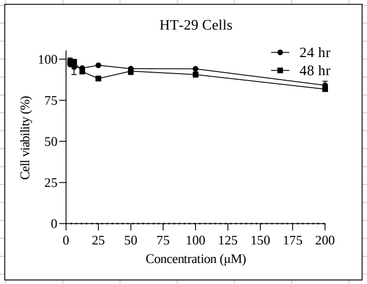

	**Control**	**TNF**	**TNF-Cin40**	**Cin40**
	1	3.6	1.819	1.195
**IL8-mRNA**	1	4.599	2.183	1.016
	1	3.031	1.292	0.91
	1	5.751	2.7981	1.19
	1	2.99	2.579	0.964
	1	3.18	1.256	1.077

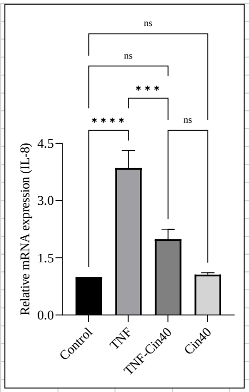

Table Anal	HT-29-IL8-mRNA
Data sets	A-D
ANOVA summary	
F	25.86
P value	<0.0001
P value su	****
Significant	Yes
R squared	0.795
Number of	1
Number of	6
Alpha	0.05

Tukey's mu	Mean Diff.	95.00% CI	Below thre	Summary	Adjusted P	Value
Control vs.	-2.859	-3.897 to -	Yes	****	<0.0001	A-B
Control vs.	-0.9879	-2.026 to 0	No	ns	0.0658	A-C
Control vs.	-0.05867	-1.097 to 0	No	ns	0.9985	A-D
TNF vs. TN	1.871	0.8323 to 2	Yes	***	0.0003	B-C
TNF vs. Ci	2.8	1.761 to 3.	Yes	****	<0.0001	B-D
TNF-Cin40	0.9292	-0.1092 to	No	ns	0.0897	C-D

Test detail	Mean 1	Mean 2	Mean Diff.	SE of diff.	n1	n2	q	DF
Control vs.	1	3.859	-2.859	0.371	6	6	10.9	20
Control vs.	1	1.988	-0.9879	0.371	6	6	3.766	20
Control vs.	1	1.059	-0.05867	0.371	6	6	0.2236	20
TNF vs. TN	3.859	1.988	1.871	0.371	6	6	7.131	20
TNF vs. Ci	3.859	1.059	2.8	0.371	6	6	10.67	20
TNF-Cin40	1.988	1.059	0.9292	0.371	6	6	3.542	20

	**Control**	**TNF**	**TNF-Cin40**	**Cin40**
	1	3.099	2.044	1.542
**CXCL1-mRN**	1	4.578	1.901	0.48
	1	3.149	2.53	0.484
	1	7.751	1.984	0.89
	1	5.778	3.187	1.004
	1	4.993	2.976	1.1124

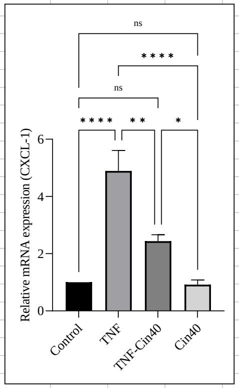

Table Anal	HT-29-CXCL1				
Data sets	A-D				
ANOVA summary					
F	23.42				
P value	<0.0001				
P value su	****				
Significant	Yes				
R squared	0.7784				
ANOVA ta	SS	DF	MS	F (DFn, DF	P value
Treatment	61.99	3	20.66	F (3, 20) =	P<0.0001
Residual (v	17.64	20	0.8822		
Total	79.63	23			
Data summary					
Number of	4				
Number of	24				
Number of	1				
Number of	6				
Alpha	0.05				

Tukey's mu	Mean Diff.	95.00% CI	Below thre	Summary	Adjusted P	Value
Control vs.	-3.891	-5.409 to -	Yes	****	<0.0001	A-B
Control vs.	-1.437	-2.955 to 0	No	ns	0.0675	A-C
Control vs.	0.08127	-1.437 to 1	No	ns	0.9988	A-D
TNF vs. TN	2.454	0.9365 to 3	Yes	**	0.0011	B-C
TNF vs. Ci	3.973	2.455 to 5.	Yes	****	<0.0001	B-D
TNF-Cin40	1.518	0.0004302	Yes	*	0.0499	C-D

Test detail	Mean 1	Mean 2	Mean Diff.	SE of diff.	n1	n2	q	DF
Control vs.	1	4.891	-3.891	0.5423	6	6	10.15	20
Control vs.	1	2.437	-1.437	0.5423	6	6	3.747	20
Control vs.	1	0.9187	0.08127	0.5423	6	6	0.2119	20
TNF vs. TN	4.891	2.437	2.454	0.5423	6	6	6.401	20
TNF vs. Ci	4.891	0.9187	3.973	0.5423	6	6	10.36	20
TNF-Cin40	2.437	0.9187	1.518	0.5423	6	6	3.959	20

HT-29 PPARγ protein expression, SiRNA treated IL8, CXCL1 mRNA:

Control	TNFα	Cin-40
0.492032	0.497793	0.674207
0.629766	0.326458	0.647492
0.79364	0.463927	0.519314
0.416385	0.166107	0.714563

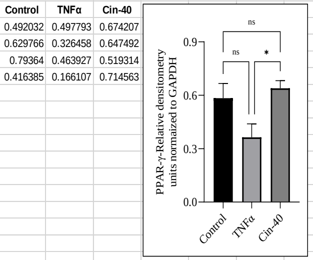

Table Anal	HT-29-Cin-PPARg-Western Blot							
Data sets	A-C							
ANOVA summary								
F	4.422							
P value	0.046							
P value su	*							
Significant	Yes							
R squared	0.4956							
ANOVA ta	SS	DF	MS	F (DFn, DF	P value			
Treatment	0.1694	2	0.08471	F (2, 9) = 4	P=0.0460			
Residual (w	0.1724	9	0.01915					
Total	0.3418	11						
Data summary								
Number of	3							
Number of	12							
Number of	1							
Number of	3							
Alpha	0.05							
Tukey's mu	Mean Diff.	95.00% CI	Below thre	Summary	Adjusted P	Value		
Control vs.	0.2194	-0.05385 to	No	ns	0.117	A-B		
Control vs.	-0.05594	-0.3292 to	No	ns	0.8382	A-C		
TNFα vs. C	-0.2753	-0.5486 to	Yes	*	0.0484	B-C		
Test detail	Mean 1	Mean 2	Mean Diff.	SE of diff.	n1	n2	q	DF
Control vs.	0.583	0.3636	0.2194	0.09786	4	4	3.17	9
Control vs.	0.583	0.6389	-0.05594	0.09786	4	4	0.8083	9
TNFα vs. C	0.3636	0.6389	-0.2753	0.09786	4	4	3.979	9

	Control	TNFα	Cin40
	1	0.647	2.624
mRNA	1	0.602	1.145
	1	0.641	1.518
	1	0.754	1.499

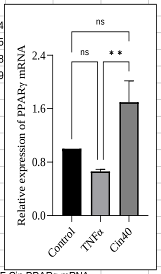

Table Anal	HT-29-TNF-Cin-PPARg-mRNA						
Data sets	A-C						
ANOVA summary							
F	8.04						
P value	0.0099						
P value su	**						
Significant	Yes						
R squared	0.6411						
ANOVA ta	SS	DF	MS	F (DFn, DF	P value		
Treatment	2.23	2	1.115	F (2, 9) = 8	P=0.0099		
Residual (w	1.248	9	0.1387				
Total	3.478	11					
Data summary							
Number of	3						
Number of	12						
Number of	1						
Number of	3						
Alpha	0.05						
Tukey's mu	Mean Diff.	95.00% CI	Below thre	Summary	Adjusted P	Value	
Control vs.	0.339	-0.3962 to	No	ns	0.4363	A-B	
Control vs.	-0.6965	-1.432 to 0	No	ns	0.0629	A-C	
TNFα vs. C	-1.036	-1.771 to -	Yes	**	0.0087	B-C	
Test detail	Mean 1	Mean 2	Mean Diff.	SE of diff.	n1	n2	q
Control vs.	1	0.661	0.339	0.2633	4	4	1.821
Control vs.	1	1.697	-0.6965	0.2633	4	4	3.741
TNFα vs. C	0.661	1.697	-1.036	0.2633	4	4	5.562

Control	PPAR-SiR	PPAR+SiRNA
1	0.23	0.44
1	0.32	0.56
1	0.18	0.39
1	0.22	0.62

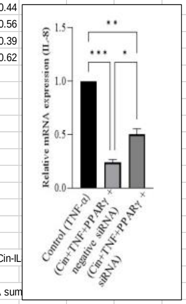

Table Anal	PPAR-g-siRNA+Cin-IL							
Repeated measures ANOVA sum								
Assume sp	No							
F	156.3							
P value	<0.0001							
P value su	****							
Statisticall	Yes							
Geisser-Gr	0.7433							
R squared	0.9812							
ANOVA ta	SS	DF	MS	F (DFn, DF	P value			
Treatment	1.199	2	0.5994	F (1.487, 4	P<0.0001			
Individual (	0.02113	3	0.007044	F (3, 6) = 1	P=0.2411			
Residual (r	0.02302	6	0.003836					
Total	1.243	11						
Data summary								
Number of	3							
Number of	4							
Number of	0							
Number of	1							
Number of	3							
Alpha	0.05							
Tukey's mu	Mean Diff.	95.00% CI	Below thre	Summary	Adjusted P	Value		
Control(TN	0.7625	0.6390 to (	Yes	***	0.0002	A-B		
Control(TN	0.4975	0.2761 to (	Yes	**	0.0053	A-C		
Column B	-0.265	-0.4554 to	Yes	*	0.0205	B-C		
Test detail	Mean 1	Mean 2	Mean Diff.	SE of diff.	n1	n2	q	DF
Control(TN	1	0.2375	0.7625	0.02955	4	4	36.5	3
Control(TN	1	0.5025	0.4975	0.05297	4	4	13.28	3
Column B	0.2375	0.5025	-0.265	0.04555	4	4	8.227	3

Control	PPAR-SiR	PPAR+SiRNA
1	0.55	0.43
1	0.45	0.26
1	0.51	0.38
1	0.41	0.43

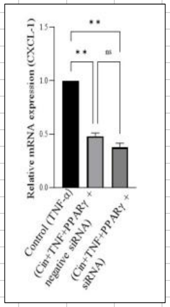

Table Anal	PPARg-SiRNA+Cin-CXCL1							
Repeated measures ANOVA summary								
Assume sp	No							
F	147.7							
P value	<0.0001							
P value su	****							
Statisticall	Yes							
Geisser-Gr	0.8688							
R squared	0.9801							
ANOVA ta	SS	DF	MS	F (DFn, DF	P value			
Treatment	0.8961	2	0.448	F (1.738, 5	P<0.0001			
Individual (	0.0127	3	0.004233	F (3, 6) = 1	P=0.3323			
Residual (r	0.0182	6	0.003033					
Total	0.927	11						
Data summary								
Number of	3							
Number of	4							
Number of	0							
Number of	1							
Number of	3							
Alpha	0.05							
Tukey's mu	Mean Diff.	95.00% CI	Below thre	Summary	Adjusted P	Value		
Column A	0.52	0.3901 to (	Yes	**	0.001	A-B		
Column A	0.625	0.4574 to (	Yes	**	0.0012	A-C		
Column B	0.105	-0.08071 to	No	ns	0.1887	B-C		
Test detail	Mean 1	Mean 2	Mean Diff.	SE of diff.	n1	n2	q	DF
Column A	1	0.48	0.52	0.03109	4	4	23.65	3
Column A	1	0.375	0.625	0.0401	4	4	22.04	3
Column B	0.48	0.375	0.105	0.04444	4	4	3.341	3

HT-29 SiRNA treated PPARγ protein and mRNA expression:

Control	PARg-siRNA
0.658262	0.175008
0.737216	0.363874
0.693641	0.214742
0.725567	0.288437

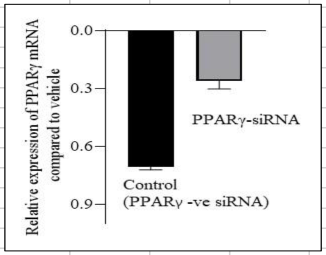

Table Anal	HT-29-PPARg-siRNA-mRNA
Column B	Data Set-B
vs.	vs.
Column A	Data Set-A
Unpaired t	test
P value	<0.0001
P value su	****
Significant	Yes
One- or tw	Two-tailed
t, df	t=9.780, df=6
How big is	the difference?
Mean of cc	0.7037
Mean of cc	0.2605
Difference	-0.4432 ± 0.04531
95% confic	-0.5540 to -0.3323
R squared	0.941
F test to	compare variances
F, DFn, Df	5.540, 3, 3
P value	0.1934
P value su	ns
Significant	No
Data analy	zed
Sample siz	4
Sample siz	4

Control	PARg-siRNA
1	0.23
1	0.14
1	0.117
1	0.192

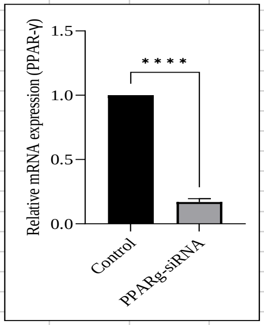

Table Anal	PPAr-g-SiRNA mRNA-Western Blot
Column B	PPARg-siRNA
vs.	vs.
Column A	Control
Unpaired t	test
P value	<0.0001
P value su	****
Significant	Yes
One- or tw	Two-tailed
Welch-cor	t=32.58, df=3.000
How big is	the difference?
Mean of cc	1
Mean of cc	0.1698
Difference	-0.8303 ± 0.02548
95% confic	-0.9113 to -0.7492
R squared	0.9972
F test to	compare variances
F, DFn, Df	Infinity, 3, 3
P value	<0.0001
P value su	****
Significant	Yes
Data analy	zed
Sample siz	4
Sample siz	4

PPARγ Promoter assay (12 h & 24 h):

No vector con	PPRE	PPRE-Cin40	PPRE-GW1929	PPRE-GW9662
551	618	2520	1558	613
240	769	1915	1420	951
360	713	2103	3344	729
272	744	1833	1961	986
29	542	1559	1164	705
633	852	1874	1527	616

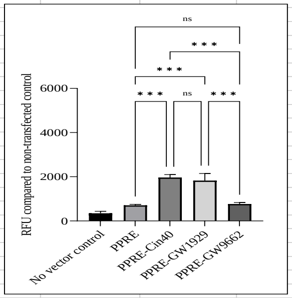

Table Anal	HT-29-PPARg-Promoter assay-12hr
Data sets	A-E
ANOVA summary	
F	19.6
P value	<0.0001
P value su	****
Significant	Yes
R squared	0.7582
Brown-Forsythe test	
F (DFn, DF	1.192 (4, 25)
P value	0.3388
P value su	ns
Are SDs s	No
Bartlett's test	
Bartlett's s	21.7
P value	0.0002
P value su	***
Are SDs s	Yes

ANOVA ta	SS	DF	MS	F (DFn, DFd)	P value
Treatment	12679913	4	3169978	F (4, 25) = 19.60	P<0.0001
Residual (v	4043694	25	161748		
Total	16723607	29			

Data summary	
Number of	5
Number of	30
Number of	1
Number of	10
Alpha	0.05

Tukey's mu	Mean Diff.	95.00% CI of diff	Below threshold?	Summary	Adjusted P Value	
No vector c	-358.8	-1041 to 323.1	No	ns	0.5442	A-B
No vector c	-1620	-2302 to -937.9	Yes	****	<0.0001	A-C
No vector c	-1482	-2163 to -799.6	Yes	****	<0.0001	A-D
No vector c	-419.2	-1101 to 262.8	No	ns	0.3929	A-E
PPRE vs.	-1261	-1943 to -579.1	Yes	***	0.0001	B-C
PPRE vs.	-1123	-1805 to -440.7	Yes	***	0.0005	B-D
PPRE vs.	-60.33	-742.3 to 621.6	No	ns	0.9989	B-E
PPRE-Cin	138.3	-543.6 to 820.3	No	ns	0.9745	C-D
PPRE-Cin	1201	518.7 to 1883	Yes	***	0.0002	C-E
PPRE-GW	1062	380.4 to 1744	Yes	***	0.001	D-E

Test detail	Mean 1	Mean 2	Mean Diff.	SE of diff.	n1	n2	q	DF
No vector c	347.5	706.3	-358.8	232.2	6	6	2.185	25
No vector c	347.5	1967	-1620	232.2	6	6	9.866	25
No vector c	347.5	1829	-1482	232.2	6	6	9.023	25
No vector c	347.5	766.7	-419.2	232.2	6	6	2.553	25
PPRE vs.	706.3	1967	-1261	232.2	6	6	7.68	25
PPRE vs.	706.3	1829	-1123	232.2	6	6	6.838	25
PPRE vs.	706.3	766.7	-60.33	232.2	6	6	0.3675	25
PPRE-Cin	1967	1829	138.3	232.2	6	6	0.8425	25
PPRE-Cin	1967	766.7	1201	232.2	6	6	7.313	25
PPRE-GW	1829	766.7	1062	232.2	6	6	6.47	25

No vector con	PPRE	PPRE-Cin4	PRE-GW19	PRE-GW9662
551	1745	4845	3958	1663
478	1468	3944	4121	1781
729	1135	6471	5022	1803
523	968	3625	4195	2155
64	770	3924	4020	1473
676	1121	5958	4536	2138

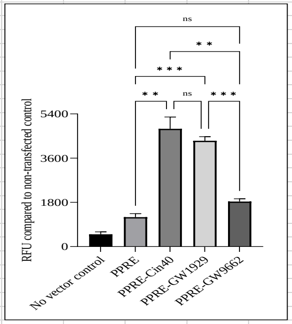

Table Anal	HT-29-PPARg-promoter assay 24 hr
Repeated measures ANOVA summary	
Assume sp	No
F	78.32
P value	<0.0001
P value su	****
Statisticall	Yes
Geisser-Gr	0.3431
R squared	0.94
Was the matching effective?	
F	2.477
P value	0.0668
P value su	ns
Is there sig	No
R squared	0.03583

ANOVA ta	SS	DF	MS	F (DFn, DF	P value
Treatment	87878622	4	21969656	F (1.373, 6	P<0.0001
Individual (	3474088	5	694818	F (5, 20) =	P=0.0668
Residual (r	5610517	20	280526		
Total	96963227	29			

Data summary	
Number of	5
Number of	6
Number of	0
Number of	1
Number of	10
Alpha	0.05

Tukey's mu	Mean Diff.	95.00% CI	Below thre	Summary	Adjusted P Value	
No vector c	-697.7	-1238 to -1	Yes	*	0.0182	A-B
No vector c	-4291	-5988 to -2	Yes	***	0.0009	A-C
No vector c	-3805	-4305 to -3	Yes	****	<0.0001	A-D
No vector c	-1332	-1682 to -9	Yes	***	0.0001	A-E
PPRE vs.	-3593	-5551 to -1	Yes	**	0.0039	B-C
PPRE vs.	-3108	-4075 to -2	Yes	***	0.0003	B-D
PPRE vs.	-634.3	-1393 to 12	No	ns	0.0947	B-E
PPRE-Cin	485.8	-952.7 to 1	No	ns	0.6758	C-D
PPRE-Cin	2959	1046 to 48	Yes	**	0.0083	C-E
PPRE-GW	2473	1816 to 31	Yes	***	0.0001	D-E

Test detail	Mean 1	Mean 2	Mean Diff.	SE of diff.	n1	n2	q	DF
No vector c	503.5	1201	-697.7	134.7	6	6	7.323	5
No vector c	503.5	4795	-4291	423	6	6	14.35	5
No vector c	503.5	4309	-3805	124.7	6	6	43.16	5
No vector c	503.5	1836	-1332	87.3	6	6	21.58	5
PPRE vs.	1201	4795	-3593	488	6	6	10.41	5
PPRE vs.	1201	4309	-3108	241.1	6	6	18.23	5
PPRE vs.	1201	1836	-634.3	189.2	6	6	4.741	5
PPRE-Cin	4795	4309	485.8	358.6	6	6	1.916	5
PPRE-Cin	4795	1836	2959	476.8	6	6	8.776	5
PPRE-GW	4309	1836	2473	163.8	6	6	21.36	5
